# Two Novel Sesquiterpenoid Glycosides from the Rhizomes of *Atractylodes lancea*

**DOI:** 10.3390/molecules27185753

**Published:** 2022-09-06

**Authors:** Lanying Liu, Fuqin Guan, Yu Chen, Fan Wang, Pengxu Chen, Min Yin, Bi Wang, Linwei Li, Qizhi Wang, Yonghua Gu, Xu Feng

**Affiliations:** 1 National Wolfberry Engineering Technology Research Center, Ningxia Academy of Agriculture and Forestry Sciences, Yinchuan 750002, China; 2 Jiangsu Key Laboratory for the Research and Utilization of Plant Resources, Jiangsu Province Engineering Research Center of Eco-cultivation and High-value Utilization of Chinese Medicinal Materials, Institute of Botany, Jiangsu Province and Chinese Academy of Sciences, Nanjing 210014, China; 3 College of Pharmacy, Nanjing University of Chinese Medicine, Nanjing 210023, China

**Keywords:** *Atractylodes lancea*, sesquiterpenoid glycosides, secoatractylohexone A, network pharmacology

## Abstract

Secoatractylohexone A (**1**), an unprecedented secoguaiane lactone glycoside featuring 6/7 cores and dihydroxy-9-guaine-3-one 11-*O*-*β*-*d*-glucopyranoside (**2**), a 9,10-unsaturated guaiene-type glycoside possessing an uncommon scaffold, were isolated from the water-soluble portion of the ethanolic extract of *Atractylodes lancea* rhizomes together with five known compounds (**3**–**7**). The structures of **1** and **2** were elucidated on the basis of extensive spectroscopic data and application of the CD technique. The potential biological activities of secoatractylohexone A were predicted by network pharmacology in silico, the result of which indicated that secoatractylohexone A may be used to treat type II diabetes.

## 1. Introduction

The genus *Atractylodes*, belonging to the Asteraceae family, consists of about seven species distributed widely in Eastern Asia and among them, five species are found in China. *Atractylodes lancea* (Thunb.) DC., which is a perennial herb known as “Cangzhu”, has been reputed in Traditional Chinese Medicine for “strengthening spleen, removing cold, and improving eyesight” [1]. The root of *A. lancea* shows therapeutic potential for treating maladies such as rheumatic diseases, digestive disorders, night blindness, and influenza [1]. The plant is mainly distributed in the provinces of Jiangsu, Hubei, and Henan, China [1].

Previous phytochemical investigations into *A. lancea* showed that this plant consisted of a series of sesquiterpenoids [2,3,4,5,6,7,8,9], polyacetylenes [6,10,11,12], saccharides [13], phenolic acids, and steroids [14], among which sesquiterpenoids were considered as characteristic chemical constituents from *A. lancea*. According to the skeleton type, sesquiterpenoids isolated from the Atractylodis rhizome can be mainly divided into three types: guaiacane, cineole, and spirosane sesquiterpenes and their glycosides [14]. Research into these sesquiterpenoids over the past two decades have shown that they exhibit various biological properties such as anti-inflammatory [15,16], anti-cancer [17], neuroprotective [18], hepatoprotective [19], and anti-diabetic activities [20].

Network pharmacology combines systems biology with multidirectional pharmacology, which is based on high-throughput omics data analysis and network database retrieval [21,22]. It has become a promising approach for domestic and foreign scholars to research the mechanism of action and predict the treatment of disease with herbs [23].

As part of our ongoing search for structurally intriguing bioactive sesquiterpenoids of *A. lancea* [3,4], secoatractylohexone A (**1**), featuring an unprecedented secoguaiane lactone glycoside skeleton with a 6/7 ring system, dihydroxy-9-guaine-3-one 11-*O*-*β*-*d*-glucopyranoside (**2**), featuring an uncommon scaffold, and five known guaiene-type compounds (**3**–**7**) were characterized. Presented herein are the isolation, structural elucidation, and potential biological activity prediction of the isolated compounds.

## 2. Results and Discussion

### 2.1. Structural Elucidation of the Isolated Compounds

**Figure 1:**
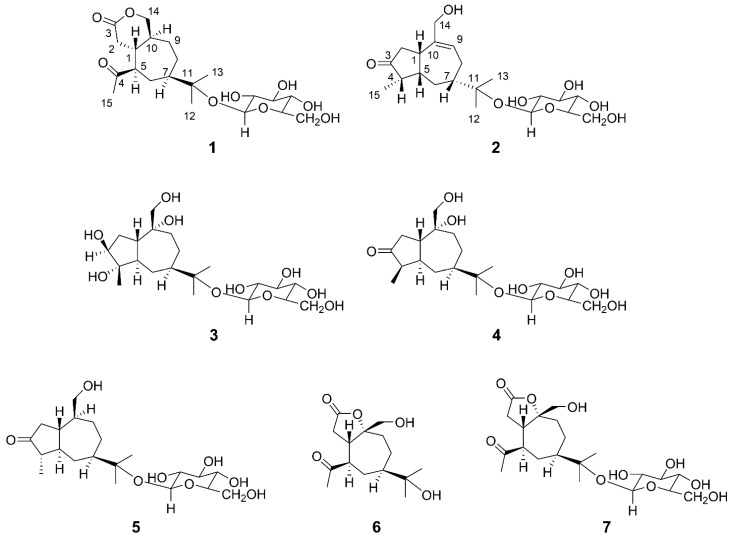
The chemical structures of compounds **1**–**7**.

The fresh rhizomes of A. lancea were extracted with ethanol (95%) at room temperature. The combined extracts were concentrated to afford a crude extract, which was suspended in water and portioned with ethyl acetate (EtOAC), and the aqueous layer was chromatographed on non-polar macroporous adsorbent resin D101 to give water and ethanol eluate fractions. The ethanol eluate fraction was chromatographed on silica gel and subjected to a combination of RP-18 (YMC; 12 nm) and Sephadex LH-20 column chromatography and semi preparative RP-HPLC to afford two new sesquiterpenoid glycosides (**1** and **2**) as well as five known ones (Figure 1). The structures of the known compounds were identified by a comparison of their NMR spectroscopic data with those reported in the literature. Their structures were identified as atractyloside B (**3**) [24,25], (1*S*,4*S*,5*S*,7*R*,10*R*)-10,11,14-trihydroxyguai-3-one 11-*O*-*β*-*d*-glucopyranoside (**4**), (1*S*,4*S*,5*R*,7*R*,10*R*)-11,14-dihydroxyguai-3-one 11-*O*-*β*-*d*-glucopyranoside (**5**), (1*S*,5*R*,7*R*,10*R*)-seco-atractylolactone (**6**), and (1*S*,5*R*,7*R*,10*R*)-seco-atractylolactone 11-*O*-*β*-*d*-glucopyranoside (**7**), respectively [25].

Compound **1** was isolated as a white amorphous powder with a positive optical rotation ${\left[\alpha \right]}_{D}^{22}$ +2.1 (*c* 0.28, MeOH). The positive ESI-HRMS provides [M+Na]^+^ and [2M+Na]^+^ peaks at *m*/*z* 453.2067 (calcd for [M+Na]^+^ 453.2101) and 883.4214, corresponding to a molecular formula of C_21_H_34_O_9_, with five degrees of unsaturation. TLC analysis displayed a yellow spot with the spraying of 1% vanillin-H_2_SO_4_ reagent, followed by heating at 105 °C after 1–2 min, and a positive reaction of the Molish reagent.

The NMR and HSQC spectral data of **1** (Table 1) showed the presence of two tert-methyls, five methylenes (one of which is connected to an oxygen atom), four methines, one hydrolated quaternary carbon, one carbonyl carbon, and one acetyl group, in addition to the β-glycopyranosyl moiety. Acid hydrolysis and gas chromatographic analysis of the persilanizated derivative of the sugar indicate that the sugar was d-glucopyranose.

Several correlations were observed in HMBC spectrum such as H-14ax (*δ* 3.76, m) with C-1 (*δ* 40.3), C-3 (*δ* 170.3), and C-10 (*δ* 35.6); H-1 (*δ* 2.21, m) with C-2 (*δ* 36.0), C-4 (*δ* 211.2), C-5 (*δ* 59.8), C-9 (*δ* 27.0), C-10 (*δ* 35.6), and C-14 (*δ* 73.7); H-2ax (*δ* 2.48, dd) with C-1 (*δ* 40.3), C-3 (*δ* 170.3), C-5 (*δ* 59.8), and C-10 (*δ* 35.6); H-5 (*δ* 3.06, m) with C-1 (*δ* 40.3), C-2 (*δ* 26.0), C-4 (*δ* 211.2), C-6 (*δ* 28.3), C-7 (*δ* 46.2), and C-10 (*δ* 35.6); H-10 (*δ* 1.69, m) with C-8 (*δ* 26.9) and C-14 (*δ* 73.7). However, the correlation between C-3 (*δ* 170.3) and 15-CH_3_ (*δ* 2.30, s), H-5 (*δ* 3.06, m) was not detected. Based on the information from the HBMC spectrum, it can be speculated that there was no direct bond between C-3 and C-4; and C-3, C-14 participated in the formation of a six-membered δ-lactone ring. Furthermore, the position of the glucosyl group was located at C-11 by the observed cross peak between the glucosyl H-1′ (*δ* 5.03, d, *J* = 7.7 Hz) and C-11 (*δ* 80.3) in the HMBC spectrum. Therefore, compound **1** can be defined as a novel secoguaiane-type glucoside with a six membered *δ*-lactone.

**Figure 2:**
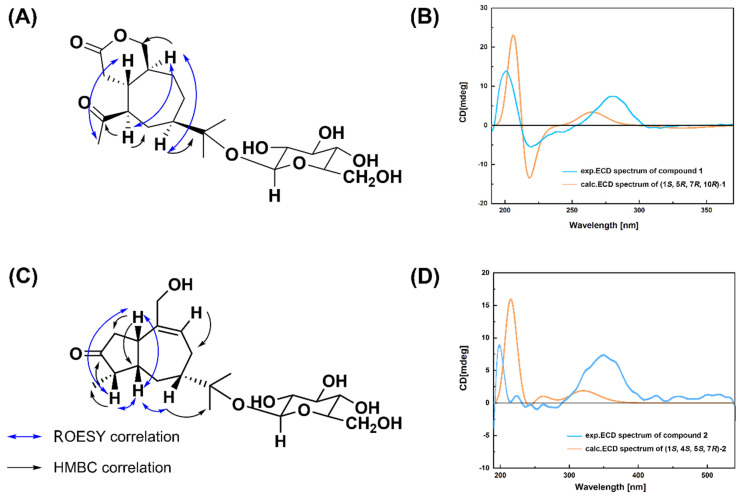
The key 2D NMR correlations and the ECD spectra of compound **1** (**A**,**B**) and compound **2** (**C**,**D**).

The relative stereochemistry of **1** was examined by the results of its ROESY spectrum. The ROESY interactions between H-1 and H-15 suggest the configuration of H-1 and H-15 should be *β*, while that between H-10 and H-5, H-7 showed that the configuration of the H-5 and H-7 were α, respectively. The relative configuration of **1** was therefore determined as (1S*,5R*,7R*,10R*), resulting in a molecular structure as shown in Figure 2A.

The absolute configuration of compound **1** was assigned by a comparison of the measured and simulated electronic circular dichroism (ECD) spectra (ECD was calculated at the TD-DFT/CAM-B3LYP/TZVP level). As shown in Figure 2B, the calculated ECD curve of (1*S*,5*R*,7*R*,10*R*)-**1** reproduced the sign and intensity of the experimentally determined positive cotton effect (CE) at 216 and 287 nm, and the negative CE at 219 nm. Therefore, the absolute configuration of **1** was assigned to (1*S*,5*R*,7*R*,10*R*). The trivial name secoatractylohexone A was given to compound **1**, and its description is (1*R*,5*S*,7*R*,10*R*)-secoatractylo-*δ*-lactone 11-*O*-*β*-*d*-glucopyranoside. Compound **1** has an unexampled secoguaiane skeleton featuring a six-membered lactone ring. Searching the literature, it was found that no compound similar to the skeleton has been reported.

Up to now, numerous guaiene-type compounds have been described in nature, but there have only been a few reports of secoguaiane-type compounds. (1*S*,5*R*,7*R*,10*R*)-seco-atractylolactone and (1*S*,5*R*,7*R*,10*R*)-seco-atractylolactone 11-*O*-*β*-*d*-glucopyranoside [25], two novel secoguaiane-type sesquiterpenoids featuring a five membered *δ*-lactone, from Atractylodes japoniea were reported, representing an uncommon secoguaiane-type with a 5/7 ring system lactone skeleton. In the present study, secoatractylohexone A (**1**) was defined as a novel secoguaiane skeletal class that possesses an unprecedented 6/7 core *δ*-lactone structure. Our discovery of **1** provides a new member to the type of secoguaiane.

Compound **2** was isolated as a white amorphous powder with a positive optical rotation [α${]}_{D}^{22}$+25.8 (*c* 0.12, MeOH). Its molecular formula of C_21_H_34_O_8_ was based on the [M+Na]^+^, [2M+Na]^+^, [M-C_6_H_12_O_6_+H]^+^ peak at *m*/*z* 437.2124 (calcd for [M+Na]^+^ 437.2151), 851.4292, 235.1697, respectively, in its ESI-HRMS spectrum, with five degrees of unsaturation. The TLC analysis displayed a cyan spot with the spraying of the 1% vanillin-H_2_SO_4_ reagent followed by heating at 105 °C after 1–2 min, and a positive reaction of the Molish reagent.

The ^1^H and ^13^C NMR spectroscopic data in combination with analysis of the DEPT and HSQC spectrum indicated the presence of a carbonyl carbon, two quaternary carbons, ten -CH-, five sp^3^ methylenes, and three methyl carbons. The ^13^C NMR spectrum showed a carbonyl at *δ* 219.1, two alkene carbons *δ* 129.9 and 143.9, and three CH3 at *δ* 12.8, 23.9, and 24.0, respectively. Careful analysis of the NMR data indicated that it was a dehydrated atractyloside derivative. Acid hydrolysis and gas chromatographic analysis of the persilanizated derivative of the sugar indicated that the sugar was d-glucopyranose.

The ^1^H NMR spectrum and ^1^H-^1^H COSY correlation spectra for **2** displayed signals for three methyl groups at *δ* 1.10 (d, 3H, *J* = 6.9 Hz, CH_3_-15), δ 1.39 (s, 3H, CH_3_-12), and *δ* 1.40 (s, 3H, CH_3_-13), and five sp^3^ methylene proton signals (-CH_2_-CH_2_-) at *δ* 2.75 (dd, 1H, *J* = 9.5, 11.8 Hz, H-2ax), 3.03 (dd, 1H, *J* = 6.1, 11.8 Hz, H-2eq); δ 1.26 (br dd, 1H, *J* = 12.6, 11.8 Hz, H-6ax), 2.61 (br d, 1H, *J* = 12.6 Hz, H-6eq); *δ* 2.05 (m, 1H, H-8ax), 2.91 (m, 1H, H-8eq); δ 4.28 (br s, 2H, CH_2_-14), and *δ* 4.31 (dd, 1H, *J* = 5.3, 11.5 Hz, H-6’ax), 4.46 (br d, 1H, *J* = 11.5 Hz, H-6’eq), respectively, and ten sp^2^ methines (Table 2) and a olefinic proton at *δ* 6.24 (d, 1H, *J* = 7.0 Hz). 

The observed ROESY interactions between H-1 and H-4, H-5, and that between H-4 and H-5, and that between H-5 and H-7, H-15 in the ROESY spectrum [Figure 2C] suggested that the ring juncture was cis H-1, H-4, H-5, H-7 were *β*. The calc. ECD pattern for (1*S*,4*S*,5*S*,7*R*)-**2** showed good accordance with the experimental ECD of **2** [Figure 2D]; therefore, **2** was characterized as (1*S*,4*S*,5*S*,7*R*)-11,14-dihydroxyl-9-guaien-3-one 11-*O*-*β*-*d*-glucopyranoside, which possesses a rare 9,10-unsaturated guaiene-type framework.

### 2.2. Network Pharmacology-Based Prediction of the Potential Biological Activity of Secoatractylohexone A

Network pharmacology is an emerging innovative method based on the rapid development of systems biology, bioinformatics, and multidirectional pharmacology to study the systematic actions and potential mechanisms of natural products [26]. In this work, the PharmMapper web server was performed to identify a total of 300 potential drug targets of secoatractylohexone A by reverse docking. The top 50 potential drug targets of secoatractylohexone A with their involving indications are listed in Table S1. The top 10 candidate targets were MTHFD1, S100A9, CTSF, CDA, UCK2, KAT2B, SULT2B1, PAH, TPSB2, and BST1. Specifically, MTHFD1, CTSF, CDA, and TPSB2 were related to treating diabetes; while SULT2B1 is mainly involved in curing kidney disease.

**Figure 3:**
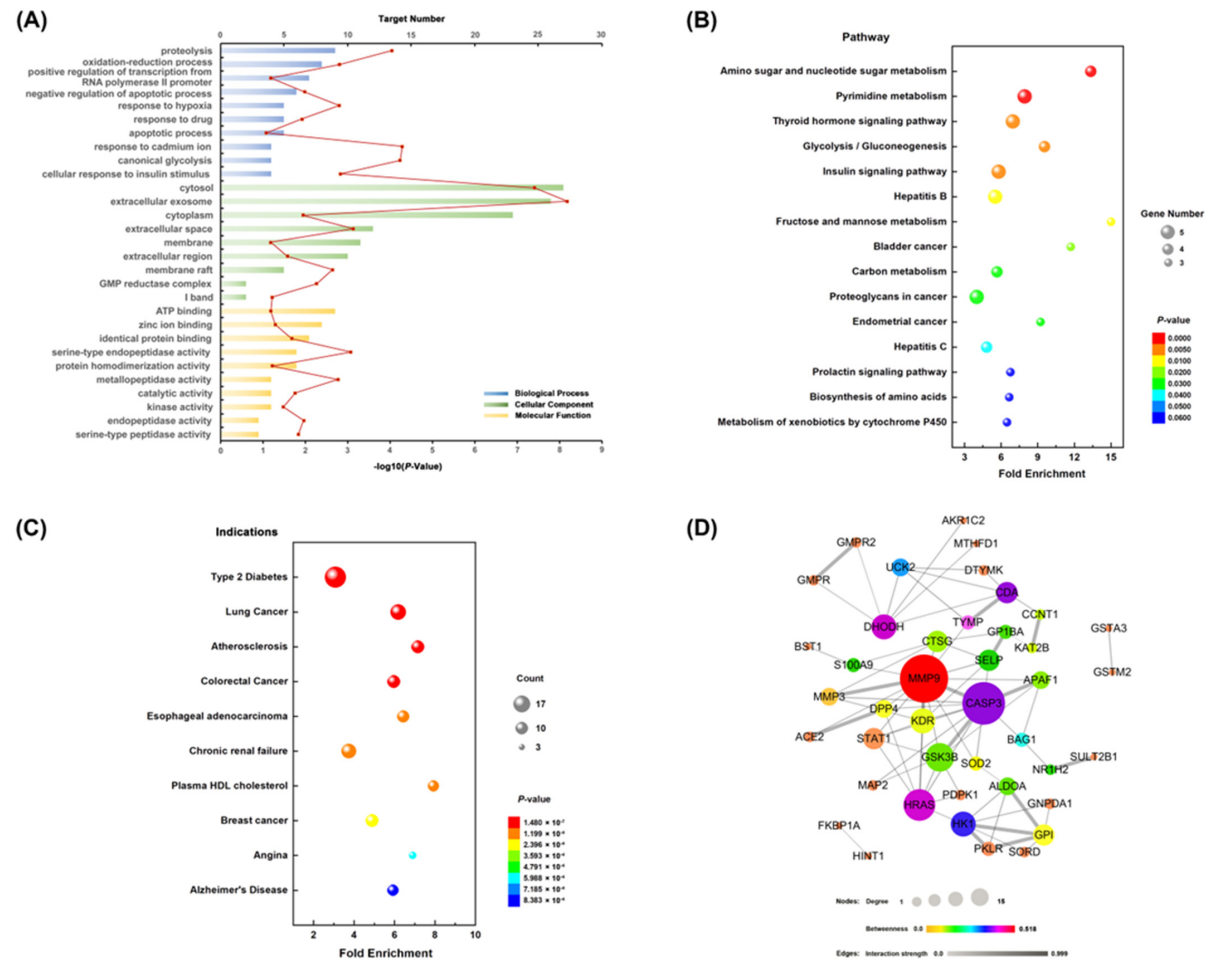
The network pharmacology analysis of secoatractylohexone A. (**A**) GO analysis of the candidate targets. Database shows the ten hub enriched items in the biological processes (BP), cell component (CC), and molecular function (MF). (**B**) The KEGG pathways of the candidate targets. (**C**) Related indications of the candidate targets. (**D**) The protein–protein interaction networks of the candidate targets.

To explore the molecular mechanism of secoatractylohexone A, the gene function (GO) and KEGG pathway enrichment of the top 50 candidate targets were uploaded into DAVID bioinformatics resources. The results of GO evaluation were illustrated by the biological process (BP), cell component (CC), and molecular function (MF) categories. In the GO analysis, 32 out of 49 BPs, seven out of nine CCs, and 10 out of 14 MFs enriched for involving targets were recognized as *p* < 0.05 (Table S2). An introduction of the GO analysis is disclosed with the top 10 enriched functions in the BP, CC, and MF terms [Figure 3A]. The GO enrichment result indicates that secoatractylohexone A regulates proteolysis, the oxidation–reduction process, the positive regulation of transcription from the RNA polymerase II promoter, canonical glycolysis, and cellular response to insulin stimulus via ATP binding, zinc ion binding, identical protein binding, and metallopeptidase activity in cytosol, extracellular exosome, cytoplasm, and extracellular space. 

Fifteen KEGG pathways with a *p*-value less than 0.07 are described in Figure 3B. Among the 15 KEGG pathways, they showed a striking functional association with the diabetes related pathways. For example, the insulin signaling pathway, glycolysis/gluconeogenesis, amino sugar and nucleotide sugar metabolism, fructose and mannose metabolism are involved in the development of diabetes. The KEGG pathway analysis also provides strong evidence that secoatractylohexone A has potential anti-diabetes biological activity. The related diseases of the top 50 targets are summarized in Figure 3C, which are mainly correlated with diabetes. 

The protein–protein interaction (PPI) network of target genes was constructed to explore the core proteins that contact other proteins in a network. As described in Figure 3D, the size and color of the circles corresponded to the interaction degree and betweenness. The center targets, which might play an important role in the biological activity of secoatractylohexone A, are matrix metalloproteinase-9 (MMP9), thymidine phosphorylase (TYMP), GTPase HRas (HRAS), dihydroorotate dehydrogenase (quinone) (DHODH), cytidine deaminase (CDA), and heat shock protein HSP 90-alpha (HSP90AA1). Investigations have shown that MMP9, TYMP, and CDA participate in many diabetes developments.

## 3. Materials and Methods

### 3.1. General Procedures

The NMR spectra were obtained with a Bruker 500 spectrometer ((Bruker, Bremen, Germany)) operating at 500 MHz for ^1^H and 125 MHz for ^13^C, respectively. Chemical shifts were reported in parts per million on the *δ* scale with TMS as the internal standard. The optical rotations were measured on a JASCO P-1020 Optical Rotation Apparatus (Jasco, Tokyo, Japan). ESI-HRMS spectra were measured on an Agilent 1100 LC/MSD TOF mass spectrometer (Agilent, California, USA). The CD spectrum were obtained on a JASCO810 spectropolarimeter (Jasco, Tokyo, Japan). Compounds **1**–**7** were purified by semi preparative HPLC (Waters, Milford, USA) using a Waters 600 liquid chromatograph with a Alltech 2000Esc ELSD detector (110 °C, flow rate of the condensed air, 3.1 mL min^−1^) and with a Phenomenex HPLC column (4 μm, 4.6 × 250 mm, Phenomenex Hydro-RP 80R). 

### 3.2. Plant Material

The rhizomes of *Atractylodes lancea* (Thunb.) DC, collected from the Maoshan Mountain in Jiangsu Province, PR China in 2019, were taxonomically identified by Prof. Chang Qi Yuan. A voucher specimen was deposited in the Nanjing Botanical Garden Mem. Sun Yat-Sen, Nanjing, Jiangsu, China (No. Atl2019).

### 3.3. Extraction and Isolation 

The fresh rhizome of A. lancea (42 kg) was extracted three times with 95% alcohol (60 L per time) at room temperature, the ethanol extract was concentrated at 50 °C under vacuum conditions, and then the residue was suspended in distilled water (10 L) to obtain an aqueous solution and successively extracted three times with ethyl acetate (10 L per time). The aqueous layer was passed through non-polar macroporous resin D-101 and eluted with water, 50% EtOH, and 90% EtOH. The 50% alcohol eluate (64 g) was chromatographed over a silica gel (200–300 mesh, 550 g, 9.0 × 75 cm) column [CHCl_3_–MeOH–H_2_O (15:1:0.05 → 10:1:0.1 → 7:3:0.5 → 2:1:0.5 → MeOH)] to give seven fractions (frs. A–G). Fraction A (1.3 g) was chromatographed over silica gel CC to give four fractions (frs.A1–A4), fraction A4 (0.15 g) was passed through silica gel CC repeatedly (EtOAc:MeOH = 10:1) to give 6 (4.0 mg). Fraction B (3.0 g) was passed through a RP-C18 column [YMC; 12 nm; MeOH: H_2_O (1:20 → 1:2)] to give six fractions (frs. B1–B6), fraction B3 (0.9 g) was subjected to Sephadex LH-20 (2 cm × 150 cm, 10% MeOH) to give three fractions (frs. B3a–B3c) and fraction B4 (0.8 g) to give two fractions (frs. B4a–B4b), fraction B3a (35 mg) was subjected to semi preparative RP-HPLC (MeOH:H_2_O = 30:70, flow rate of 2.5 mL min^-1^) to **1** (5 mg), and fraction B4b (105 mg) was subjected to repeated semi preparative RP-HPLC (MeOH:H_2_O = 33:67) to give **2** (6 mg) and **5** (14 mg). Fraction C (8.0 g) was applied to a silica gel column eluted in a step gradient manner with CHCl_3_–MeOH (from 10:1 to 4:1) to afford fractions C1–C7. Fraction C3 (4.0 g) was subjected to Sephadex LH-20 (2 cm × 150 cm, 13% MeOH) to give four fractions (C3a-C3d), C3b (1.3 g) was passed through a RP-18 column (YMC; 12 nm; MeOH: H_2_O = 1:20) to give three fractions (C3b-1-C3b-3), and C3b-1 and C3b-3 were applied to a silica column to give **4** (7.5 mg) and **7** (90 mg), respectively. Fraction F (4.9 g) was applied to a silica gel column again to afford four fractions (frs. F1–F4), and fraction F4 (1.2 g) was passed through a RP-C18 column (MeOH: H2O = 5:95) to give **3** (140 mg).

(1*S*,5*R*,7*R*,10*R*)-Secoatractylo-*δ*-lactone 11-*O*-*β*-*d*-glucopyranoside (**1**): White amorphous powder; [*α*${]}_{D}^{22}$: +2.1 (*c* 0.28, MeOH); IR (KBr) *ν*_max_, cm^−1^: 3433, 2947, 1741, 1710, 1377, 896; (+)-ESI-HRMS *m*/*z* 453.2067 [M+Na]^+^ (*calcd* for 453.2101), 883.4214 [2M+Na]^+^; ^1^H and ^13^C NMR shown in Table 1; ECD (*c* 0.01, MeOH) *λ*_max_ 206 (+), 219 (−), 287 (+) nm.

(1*S*,4*S*,5*S*,7*R*)-11,14-Dihydroxy-9-guaien-3-one 11-O-*β*-*d*-glucopyranoside (**2**): White amorphous powder; [α${]}_{D}^{22}$: +14.9 (*c* 0.12; MeOH); IR (KBr) *ν*_max_, cm^−1^: 3442, 2945, 1622, 1375, 897; (+)-ESI-HRMS *m*/*z* 437.2124 [M+Na]^+^ (calcd for 437.2151), 851.4292 [2M+Na]^+^, 235.1697 [M-C_6_H_12_O_6_+H]^+^; ^1^H and ^13^C NMR shown in Table 2; ECD (*c* 0.01, MeOH) *λ*_max_ 198 (+), 346 (+) nm. 

Atractyloside B (**3**): ^13^C-NMR (C_5_D_5_N, 125 MHz) δ 44.0 (C-1), 37.9 (C-2), 79.6 (C-3), 75.3 (C-4), 52.5 (C-5), 31.9 (C-6), 45.9 (C-7), 24.4 (C-8), 31.2 (C-9), 81.2 (C-10), 80.6 (C-11), 24.0 (C-12), 24.9 (C-13), 68.6 (C-14), 16.0 (C-15), 98.6 (C-1’), 75.8(C-2’), 78.8 (C-3’), 71.8 (C-4’), 78.2 (C-5’), 62.8 (C-6’) [25].

(1*S*,4*S*,5*S*,7*R*,10*R*)-10,11,14-Trihydroxyguai-3-one 11-O-*β*-*d*-glucopyranoside (**4**): ^13^C-NMR (C_5_D_5_N, 125 MHz) *δ* 46.7 (C-1), 39.3 (C-2), 219.4 (C-3), 52.2 (C-4), 44.1 (C-5), 36.7 (C-6), 50.8 (C-7), 23.4 (C-8), 37.0 (C-9), 74.5 (C-10), 80.3 (C-11), 23.9 (C-12), 24.4 (C-13), 69.6 (C-14), 12.9 (C-15), 98.6 (C-1’), 75.3 (C-2’), 78.9 (C-3’), 71.9 (C-4’), 78.1 (C-5’), 63.0 (C-6’) [25].

(1*S*,4*S*,5*R*,7*R*,10*R*)-11,14-Dihydroxyguai-3-one 11-O-*β*-*d*-glucopyranoside (**5**): ^13^C-NMR (C_5_D_5_N, 125 MHz) *δ* 43.6 (C-1), 45.6 (C-2), 218.8 (C-3), 52.3 (C-4), 48.9 (C-5), 35.7 (C-6), 50.1 (C-7), 25.6 (C-8), 28.8 (C-9), 48.0 (C-10), 80.4 (C-11), 24.1 (C-12), 24.2 (C-13), 66.0 (C-14), 12.6 (C-15), 98.6 (C-1’), 75.4 (C-2’), 78.9 (C-3’), 72.0 (C-4’), 78.1 (C-5’), 63.1 (C-6’) [25].

(1*S*,5*R*,7*R*,10*R*)-Seco-atractylolactone (**6**): ^13^C-NMR (C_5_D_5_N, 125 MHz) *δ* 41.1 (C-1), 37.3 (C-2), 175.9 (C-3), 209.4 (C-4), 55.5 (C-5), 32.0 (C-6), 53.9 (C-7), 25.2 (C-8), 32.6 (C-9), 91.4 (C-10), 71.4 (C-11), 26.0 (C-12), 28.5 (C-13), 68.7 (C-14), 28.6 (C-15) [25].

(1*S*,5*R*,7*R*,10*R*)-Seco-atractylolactone 11-O-*β*-*d*-glucopyranoside (**7**): ^13^C-NMR (C_5_D_5_N, 125 MHz) *δ* 41.1 (C-1), 37.4 (C-2), 175.8 (C-3), 209.9 (C-4), 55.5 (C-5), 31.8 (C-6), 52.2 (C-7), 25.2 (C-8), 32.8 (C-9), 91.4 (C-10), 79.6 (C-11), 22.7 (C-12), 25.5 (C-13), 68.7 (C-14), 28.8 (C-15), 98.9 (C-1’), 75.4 (C-2’), 79.0 (C-3’), 72.1 (C-4’), 78.3 (C-5’), 63.0 (C-6’) [25].

### 3.4. General Method for Acid Hydrolysis

Each glycoside (2 mg) was heated in 1 mL of 1 M HCl (dioxane: H_2_O, 1:1) at 80 °C for 3 h in a water bath. Dioxane was removed and the solution was extracted with EtOAc (1 mL × 3). The EtOAc portion was washed with water and removed the EtOAc. The aqueous solution of the acid hydrolysis of each glycoside was neutralized by passing through an Amberlite MB-3 resin column eluted with water, then concentrated and dried. The dried sugar mixture was dissolved in pyridine (0.5 mL), and then treated with hexamethyl-disilazane (0.2 mL) and trimethylchlorosilane (0.1 mL) at room temperature for 6 h. After centrifugation, the above fraction was analyzed by GC analysis with authentic monosaccharides.

### 3.5. Quantum Chemical Calculation

The conformational search for (1*S*,5*R*,7*R*,10*R*)-**1** and (1*S*,4*S*,5*S*,7*R*)-**2** were conducted by the Spartan 14 program [27] using a molecular mechanics force field. The conformers within a 5 kcal mol^−1^ energy window were initially optimized at the density functional theory (DFT)/Becke-3-Lee-Yang-Parr (B3LYP)/6-31G+(d) level. The frequency calculation was then conducted for the previously optimized conformers to obtain the corresponding relative Gibbs free energies (∆*G*). Boltzmann weighting factors (*P_i_*%) of each conformer were calculated on the basis of ∆*G* to eliminate the conformers that possess an inappreciable contribution. Subsequently, the conformers selected for ECD calculation were re-optimized by DFT calculations at the CAM-B3LYP/TZVP level. Then, the 20 lowest electronic transitions were calculated using the time-dependent density functional theory (TD-DFT) method at the CAM-B3LYP/TZVP level, and with methanol as the solvent employing the PCM model. All quantum chemical calculations were conducted using the Gaussian 09 program package [28]. The ECD curves were generated with SpecDis [29] at a half bandwidth of 0.38 and 0.32 eV for **1** and **2**, respectively.

### 3.6. Network Pharmacology-Based Prediction of the Potential Biological Actions of Compound **1**

The candidate targets of compound **1** were searched on the PharmMapper WebServer (http://lilab.ecust.edu.cn/pharmmapper/, accessed on 10 December 2021) with the target database limited to “Human Protein Targets Only” by the reverse docking algorithm. The screened targets were renamed to official gene names by using UniProt (http://www.uniprot.org/, accessed on 18 December 2021) with human normalization. The Database for Annotation, Visualization, and Integrated Discovery (DAVID) ver. 6.8 (https://david.ncifcrf.gov/, accessed on 18 December 2021) and the Kyoto Encyclopedia of Genes and Genomes (KEGG) pathways (http://www.genome.jp/kegg/pathway.html, accessed on 18 December 2021) were used to search for information about the functional annotation of candidate genes for extracting potential pathways that showed high accordance with these proteins. The Search Tool for the Retrieval of Interacting Genes (STRING) database (https://string-db.org/, accessed on 20 December 2021) was utilized to hunt for the protein–protein interaction between candidate targets, with the organism defined to “Homo sapiens”, a confidence score > 0.9, and interaction number < 50. The protein–protein interaction networks were constructed using Cytoscape 3.7.2. Topological parameters of the network (degree, betweenness centrality, and interaction strength) were implemented using the network analyzer plug-in in Cytoscape to judge the importance of nodes in the given network.

## 4. Conclusions

The crude extract of *Atratylodes lancea* revealed the isolation and purification of two new (**1** and **2**) and five (**3**–**7**) known compounds. Compound **1** was clarified to be (1*S*,5*R*,7*R*,10*R*)-secoatractylo-*δ*-lactone 11-*O*-*β*-*d*-glucopyranoside, named secoatractylohexone A, which has an unexampled secoguaiane skeleton featuring a six-membered lactone ring. Compound **2** has been clarified as (1*S*,4*S*,5*S*,7*R*)-11,14-dihydroxyl-9-guaien-3-one 11-*O*-*β*-*d*-glucopyranoside, which has an uncommon scaffold. Furthermore, network pharmacology predicted the treatment of diabetes with secoatractylohexone A, along with the potential targets and mechanism. According to all of these shreds of evidence, it is believed that the potency of secoatractylohexone A is attributed to its dissimilar orientation. Therefore, further studies are required to confirm the potency of secoatractylohexone A.

## Figures and Tables

**Table 1:** The ^1^H-NMR (500 MHz), ^13^C-NMR (125 MHz), HMBC, and ROESY spectra data for compound **1** (*δ*, TMS, pyridine-*d*_5_).

No.	*δ*_C_	*δ*_H_ (*J*, Hz)	HMBC	ROESY
1	40.3	2.21, m, 1H	C-2, 4, 5, 9, 10, 14	H-14, 15
2	36.0	2.48, dd (1H, 10.6, 17.8), H-2ax	C-1, 3, 5, 10	H-6, 9, 10, 15
		2.86, dd (1H, 6.1, 17.8), H-2eq		
3	170.3			
4	211.2			
5	59.8	3.06, m, 1H	C-1, 2, 4, 6, 7, 10	H-10, 15
6	28.3	1.21, m, 1H, H-6ax	C-1, 5, 7, 8	H-2, 15
		2.43, m, 1H, H-6eq	C-4, 11	
7	46.2	2.22, m, 1H	C-5, 11	H-1′, 10, 12, 13
8	26.9	1.22, m, 1H, H-8ax	C-9	H-12, 13
		1.59, m, 1H, H-8eq		
9	27.0	1.20, m, 2H	C-1, 8, 14	H-2, 14
10	35.6	1.69, m, 1H	C-8, 14	H-2eq, 5, 7
11	80.3			
12	21.7	1.16, s, CH_3_	C-3′, 5′, 11, 7, 13	H-1′, 7, 8
13	25.1	1.42, s, CH_3_	C-2′, 3′, 4′, 5′, 7, 11, 12	H-1′, 7, 8
14	73.7	3.76, dd (1H, 11.2, 11.3), H-14ax	C-1, 3, 10	H-1, 9
		4.05, dd (1H, 9.0, 11.2), H-14eq		
15	29.7	2.30, s, CH_3_	C-4, 5	H-1, 2, 5, 6
1′	98.8	5.03, d (1H, 7.7)	C-11	H-7, 12, 13
2′	75.5	3.95, m, 1H	C-1′, 3′	
3′	78.9	4.20, m, 1H	C-2′, 4′	
4′	72.1	4.06, m, 1H	C-3′, 5′, 6′	
5′	78.8	3.97, m, 1H	C-4′	
6′	63.0	4.62, dd (1H, 1.9, 11.4), H-6′ax	C-4′, 5′	
		4.24, br d (1H, 11.4), H-6′eq		

**Table 2:** The ^1^H-NMR (500 MHz), ^13^C-NMR (125 MHz), HMBC, and ROESY spectra data for Compound **2** (*δ*, TMS, pyridine-*d*_5_).

No.	*δ*_C_	*δ*_H_ (*J*, Hz)	HMBC	ROESY
1	42.1	2.91, m, 1H	C-2, 5, 9, 10	H-1, 9, 14
2	40.7	2.75, dd (1H, 9.5, 11.8), H-2ax	C-1, 3, 5, 10	H-4, 5, 8, 14
		3.03, dd (1H, 6.1,11.8), H-2eq		
3	219.1			
4	52.1	1.79, m, 1H	C-3, 5, 6, 15	H-2, 15
5	49.4	1.57, m, 1H		H-1, 2, 7, 15
6	39.4	1.26, br dd (1H, 12.6, 11.8), H-6ax	C-1, 5, 7, 8	H-8, 12, 13, 15
		2.61, br d (1H, 12.6), H-6eq		
7	46.2	1.76, m, 1H	C-6, 8, 11, 12, 13	H-1′, 5, 12, 13
8	29.4	2.05, m, 1H, H-8ax	C-6, 7, 9, 10, 11	H-2, 6, 12, 13
		2.91, m, 1H, H-8eq		
9	129.9	6.24, d, (1H, 7.0)	C-1, 8, 14	H-1, 14
10	143.9			
11	80.0			
12	23.9	1.39, s, CH_3_	C-7, 11, 13	H-1′, 6, 7, 8
13	24.0	1.40, s, CH_3_	C-7, 11, 12	H-1′, 6, 7, 8
14	66.1	4.28, br s, 2H	C-1, 9, 10	H-1, 2, 9
15	12.8	1.10, d (3H, 6.9), CH_3_	C-3, 4, 5	H-4, 5, 6
1′	98.6	4.99, d (1H, 7.7)	C-11, 3′	H-7, 12, 13
2′	75.3	3.97, dd (1H, 8.0, 7.8)	C-1′, 3′	2′-OH
3′	78.9	4.21, dd ( 1H, 8.6, 7.7)	C-2′, 4′	
4′	71.9	4.18, dd (1H, 7.7, 8.7)	C-3′, 5′	
5′	78.0	3.87, m, 1H	C-4′	
6′	63.0	4.31, dd (1H, 5.3, 11.5), H-6′ax	C-4′, 5′	
		4.46, br d (1H, 11.5), H-6′eq		

The HMBC spectrum [Figure 2C] showed a long-range correlation between H-4 (*δ* 1.79, m) with C-3 (*δ* 219.1), C-6 (*δ* 39.4), and C-15 (*δ* 12.8); and H-15 (*δ* 1.10, d, *J* = 6.9 Hz) with C-3 (*δ* 219.1), C-4 (*δ* 52.1), C-5 (*δ* 49.4).

## Data Availability

All the data in this research are presented in manuscript and Supplementary Material.

## Supplementary Materials

The following supporting information can be downloaded at: https://www.mdpi.com/article/10.3390/molecules27185753/s1, Figure S1: ESI-HRMS spectrum compound of **1**; Figure S2: 13C-NMR spectrum of compound **1**; Figure S3: 1H-NMR spectrum of compound **1**; Figure S4: HSQC spectrum of compound **1**; Figure S5: HMBC spectrum of compound **1**; Figure S6: ROESY spectrum of compound **1**; Figure S7: CD spectrum of compound **1**; Figure S8: ESI-HRMS spectrum of compound **2**; Figure S9: 13C-NMR spectrum of compound **2**; Figure S10: 1H-NMR spectrum of compound **2**; Figure S11: HSQC spectrum of compound **2**; Figure S12: HMBC spectrum of compound **2;** Figure S13: ROESY spectrum of compound **2**; Figure S14: CD spectrum of compound **2**; Figure S15: 13C-NMR spectrum of compound **3**; Figure S16: 13C-NMR spectrum of compound **4**; Figure S17: 13C-NMR spectrum of compound **5**; Figure S18: 13C-NMR spectrum of compound **6**; Figure S19: 13C-NMR spectrum of compound **7**; Table S1. The top 50 potential drug targets of compound **1**; Table S2. The gene function analysis of the top 50 candidate targets for compound **1**; Table S3. The KEGG pathway enrichment of the top 50 candidate targets for compound **1**; Table S4. The top 10 involved indications of the top 50 candidate targets for compound **1**.

## Sample Availability

Samples of the compounds **3** and **7** are available from the authors.
