# Supplementary material for: Two Novel Sesquiterpenoid Glycosides from the Rhizomes of Atractylodes lancea
Source: Molecules. 2022 Sep 6;27(18):5753. doi: 10.3390/molecules27185753 (PMC9500871; doi:10.3390/molecules27185753)
Supplement: Supplementary file 1 [file molecules-27-05753-s001.zip › molecules-1887545-SI.pdf]

Two novel sesquiterpenoid glycosides from the Rhizomes of *Atractylodes lancea* (Thunb.) DC.

Lanying Liu [1,†], Fuqin Guan [2,†], Yu Chen [2], Fan Wang [2,3], Pengxu Chen [2,3], Min Yin [2], Bi Wang [2], Linwei Li [2,*], Qizhi Wang [2,*], Yonghua Gu [2,*] and Xu Feng [2,3]

[1] National Wolfberry Engineering Technology Research Center, Ningxia Academy of Agriculture and Forestry Sciences, Yinchuan 750002, China
[2] Jiangsu Key Laboratory for the Research and Utilization of Plant Resources, Jiangsu Province Engineering Research Center of Eco-cultivation and High-value Utilization of Chinese Medicinal Materials, Institute of Botany, Jiangsu Province and Chinese Academy of Sciences, Nanjing 210014, China
[3] College of Pharmacy, Nanjing University of Chinese Medicine, Nanjing 210023, China
* Correspondence: xt_llw@163.com (L.L.); wangqizhi@cnbg.net (Q.W.); guyhua@cnbg.net (Y.G.)
† These authors contributed equally to this work.

1. **Figure S1** ESI-HRMS spectrum compound of **1**

2. **Figure S2** ^{13}C-NMR spectrum of compound **1**

3. **Figure S3** ^{1}H-NMR spectrum of compound **1**

4. **Figure S4** HSQC spectrum of compound **1**

5. **Figure S5** HMBC spectrum of compound **1**

6. **Figure S6** ROESY spectrum of compound **1**

7. **Figure S7** CD spectrum of compound **1**

8. **Figure S8** ESI-HRMS spectrum of compound **2**

9. **Figure S9** ^{13}C-NMR spectrum of compound **2**

10. **Figure S10** ^{1}H-NMR spectrum of compound **2**

11. **Figure S11** HSQC spectrum of compound **2**

12. **Figure S12** HMBC spectrum of compound **2**

13. **Figure S13** ROESY spectrum of compound **2**

14. **Figure S14** CD spectrum of compound **2**

15. **Figure S15** ^{13}C-NMR spectrum of compound **3**

16. **Figure S16** ^{13}C-NMR spectrum of compound **4**

17. **Figure S17** ^{13}C-NMR spectrum of compound **5**

18. **Figure S18** ^{13}C-NMR spectrum of compound **6**

19. **Figure S19** ^{13}C-NMR spectrum of compound **7**

20. **Table S1.** The top 50 potential drug targets of compound **1**

21. **Table S2.** The gene function analysis of top 50 candidate targets for compound **1**

22. **Table S3.** KEGG pathway enrichment of top 50 candidate targets for compound **1**

23. **Table S4.** The top 10 involved indications of top 50 candidate targets for compound **1**

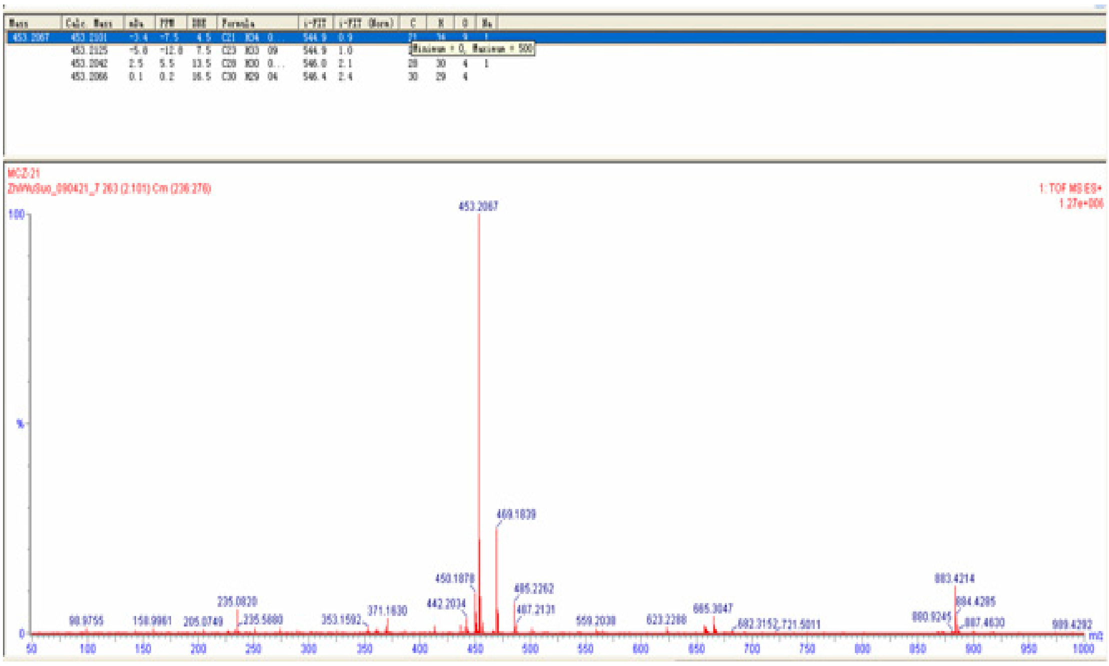

Figure S1 ESI-HRMS spectrum compound of **1**

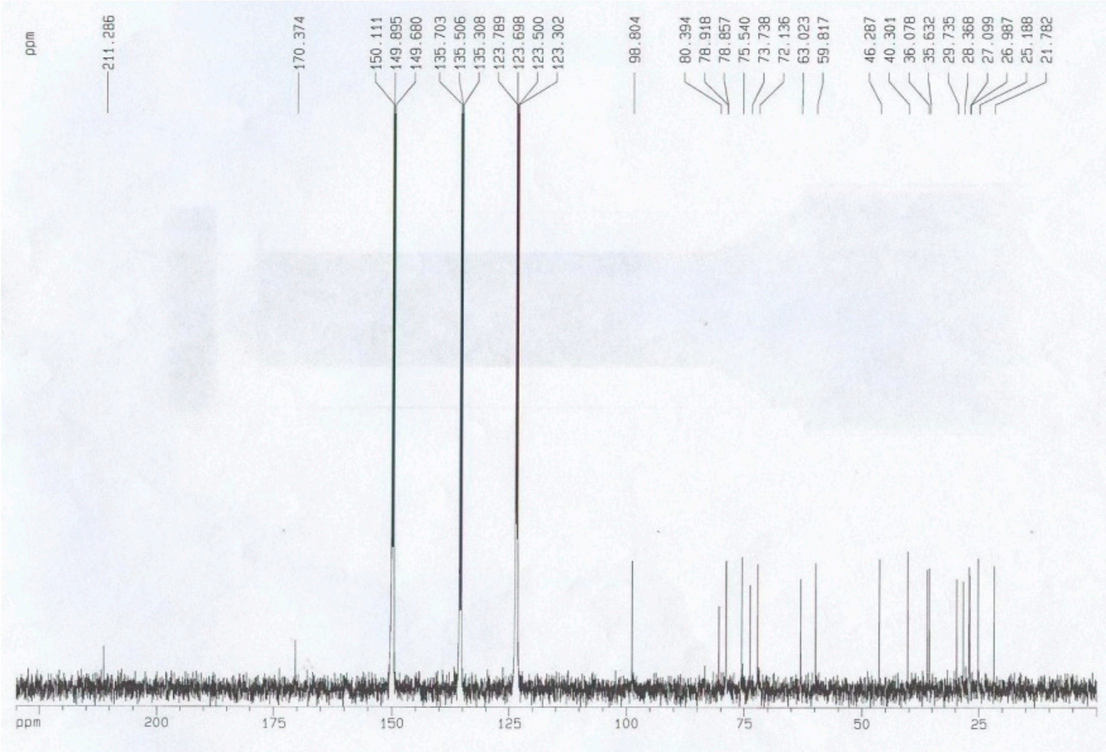

Figure S2 ^{13}C-NMR spectrum of compound **1**

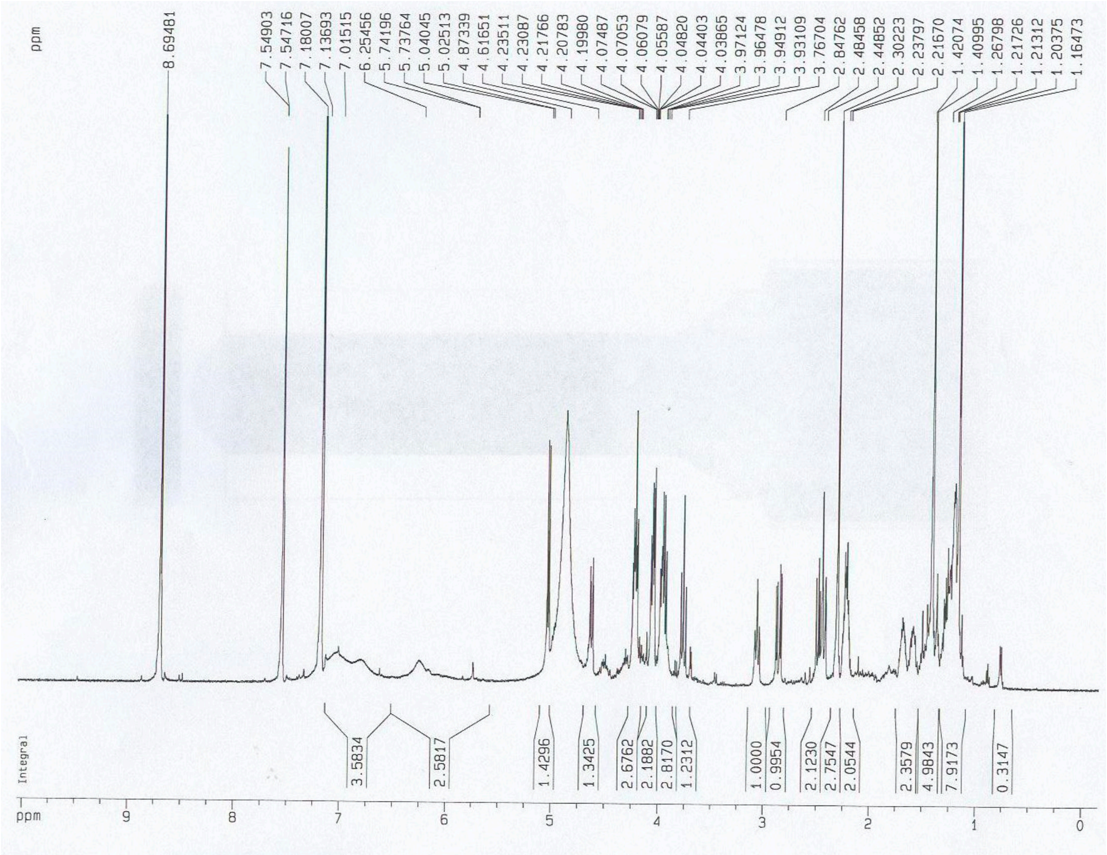

Figure S3 ^{1}H-NMR spectrum of compound **1**

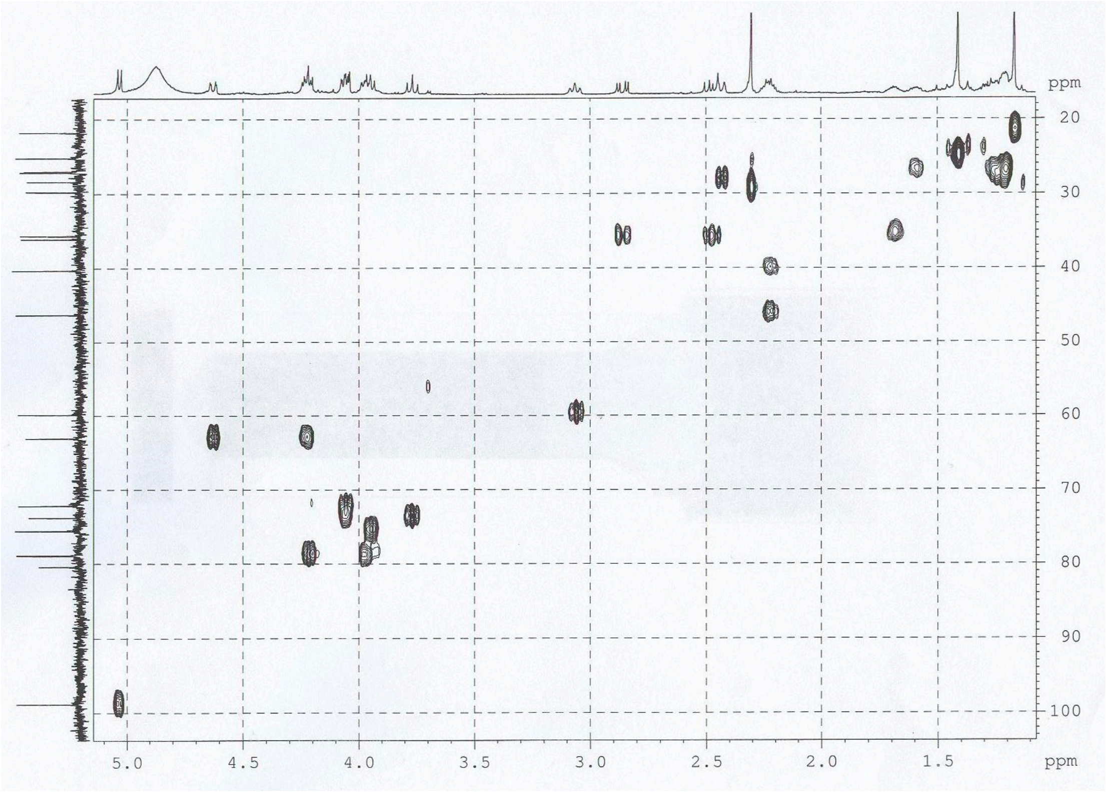

Figure S4 HSQC spectrum of compound **1**

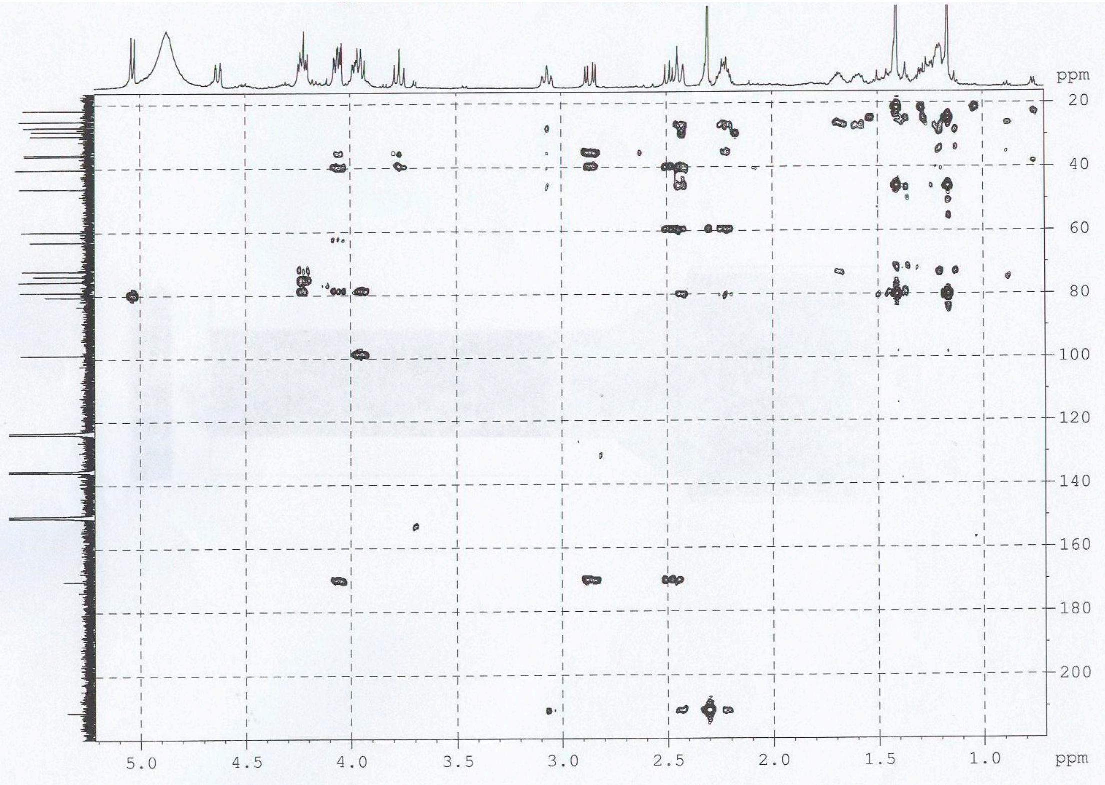

Figure S5 HMBC spectrum of compound **1**

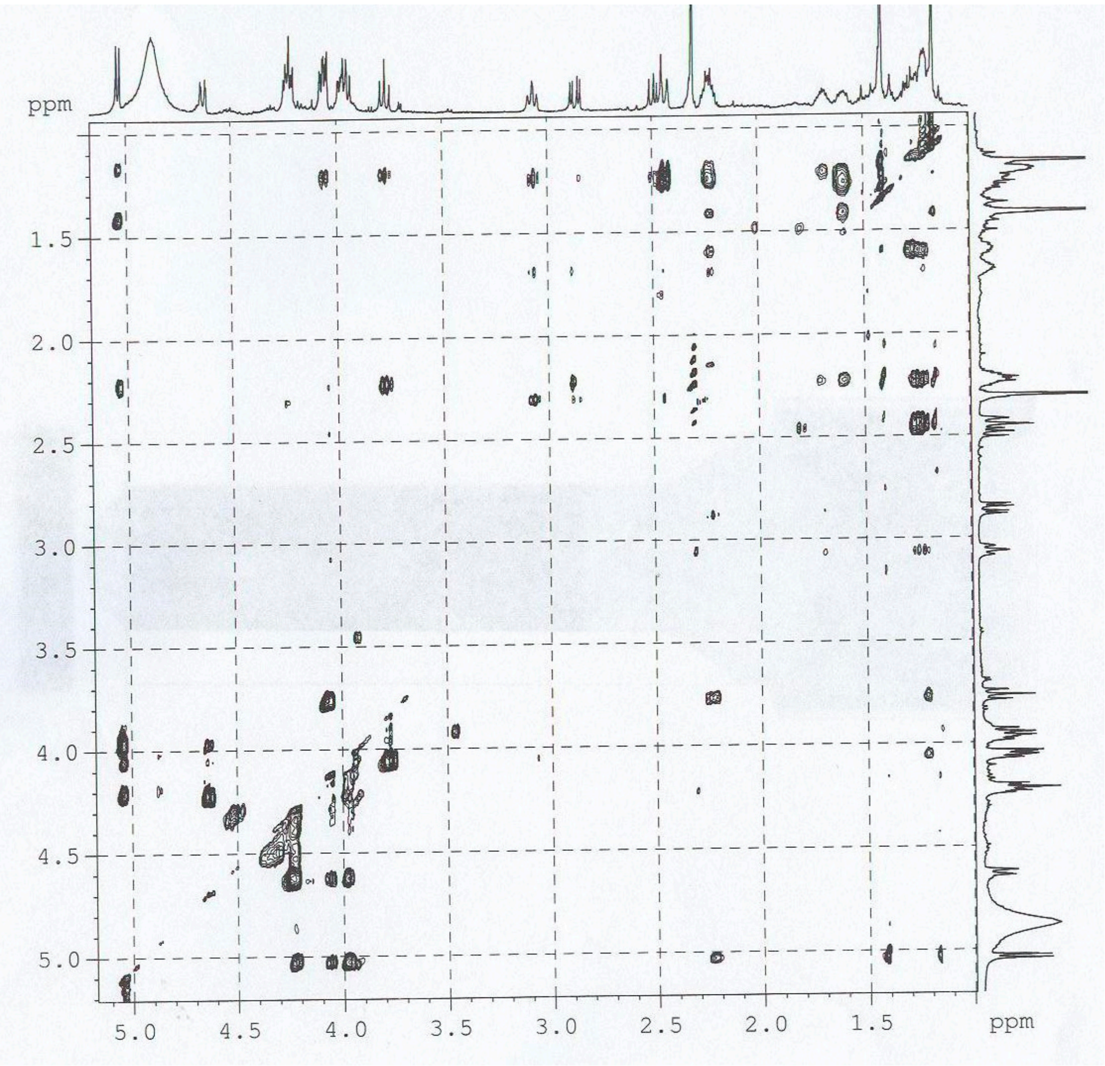

Figure S6 ROESY spectrum of compound **1**

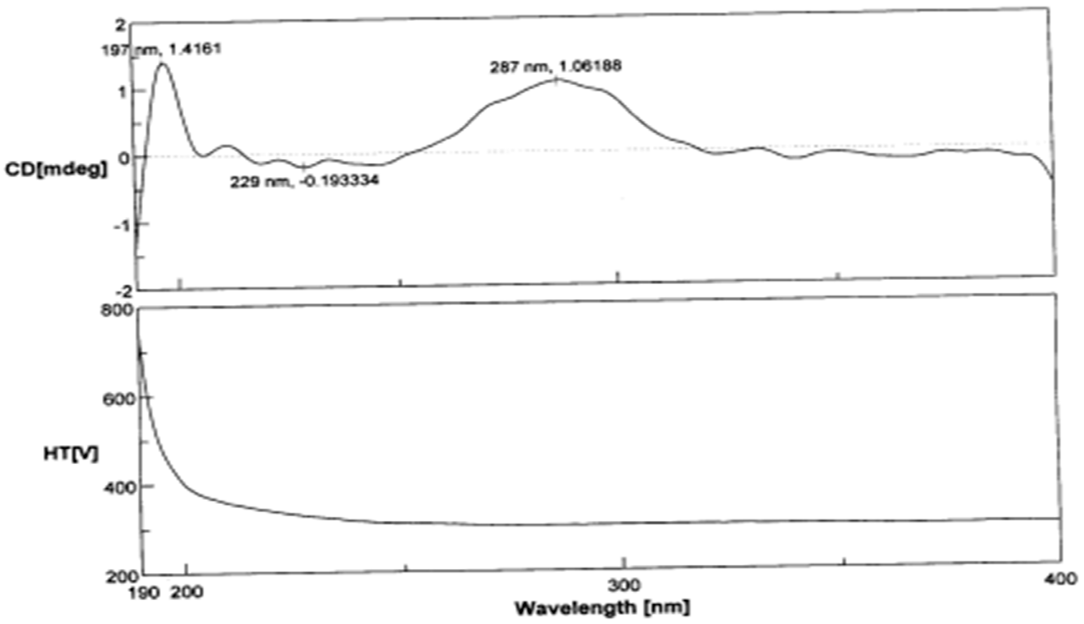

Date	2009-5-18 15:24
File name	MCZ-21s.jws
Model	J-810
Serial No.	B027760750
Band width	1 nm
Response	1 sec
Sensitivity	Standard
Measurement range	400 - 190 nm
Data pitch	1nm
Scanning speed	100 nm/min
Accumulation	3
Cell Length	0.05 cm
Concentration	1 (w/v)%
Solvent	MeOH
Temperature	Room Temperature
Sample name	MCZ-21
Operator	Lin
Comment	

Figure S7 CD spectrum of compound **1**

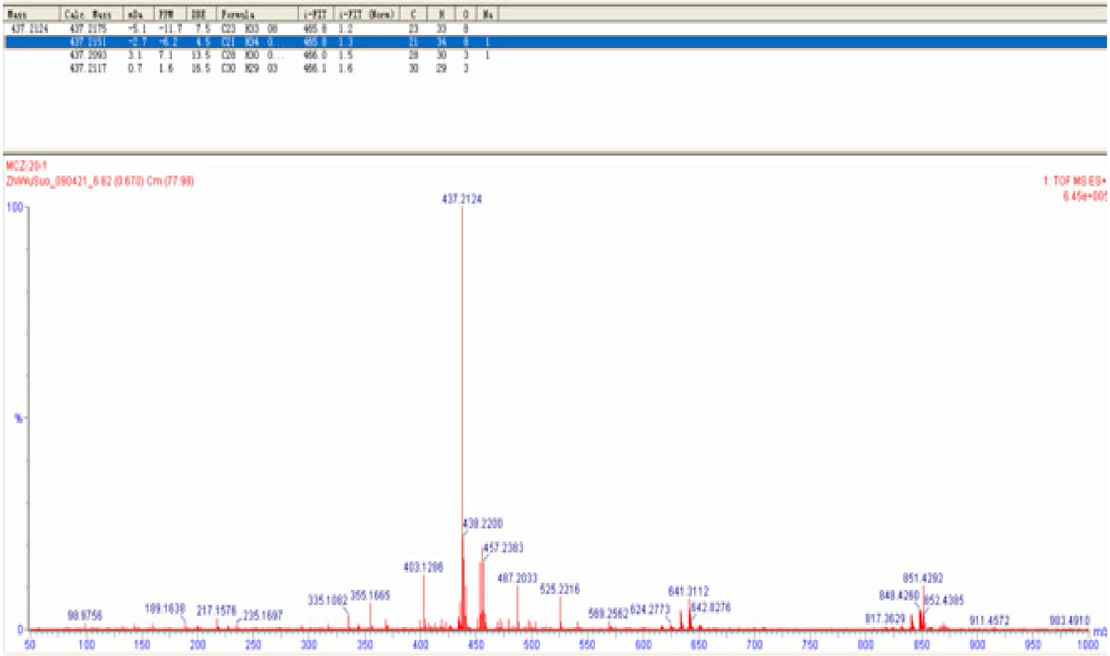

Figure S8 ESI-HRMS spectrum of compound **2**

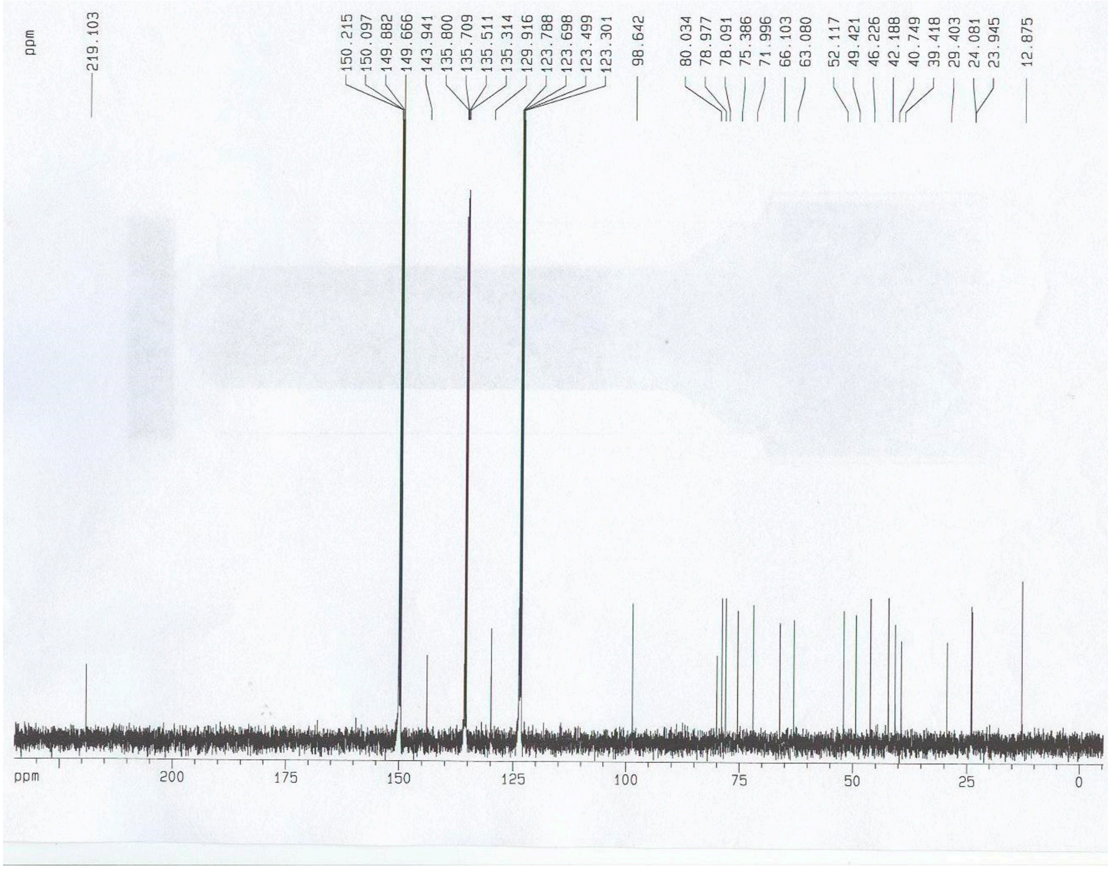

Figure S9 ^{13}C-NMR spectrum of compound **2**

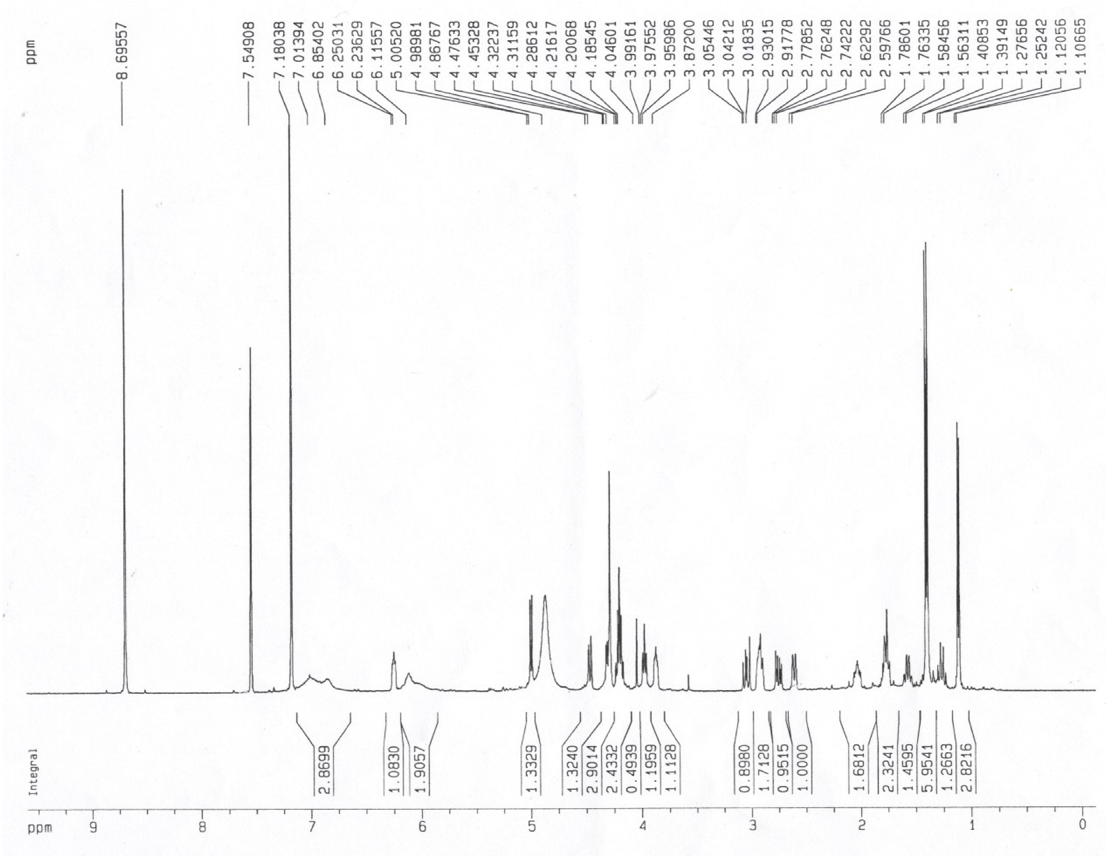

Figure S10 ^{1}H-NMR spectrum of compound **2**

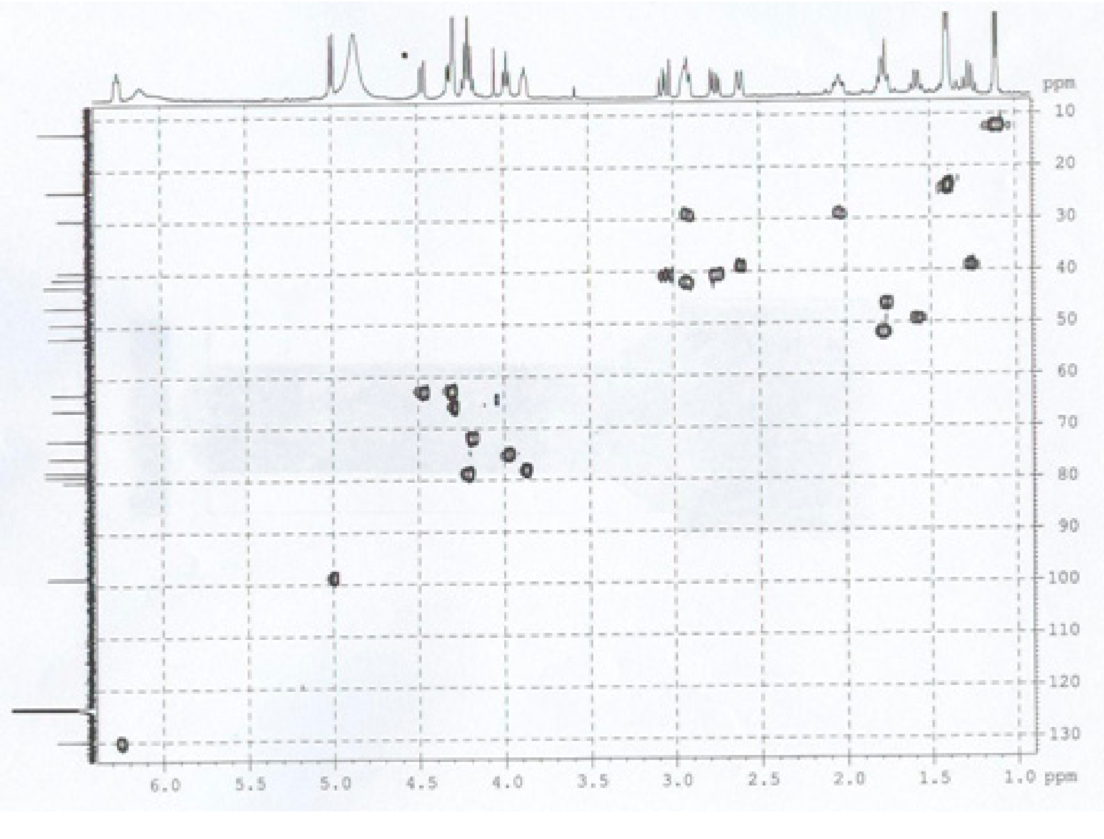

Figure S11 HSQC spectrum of compound **2**

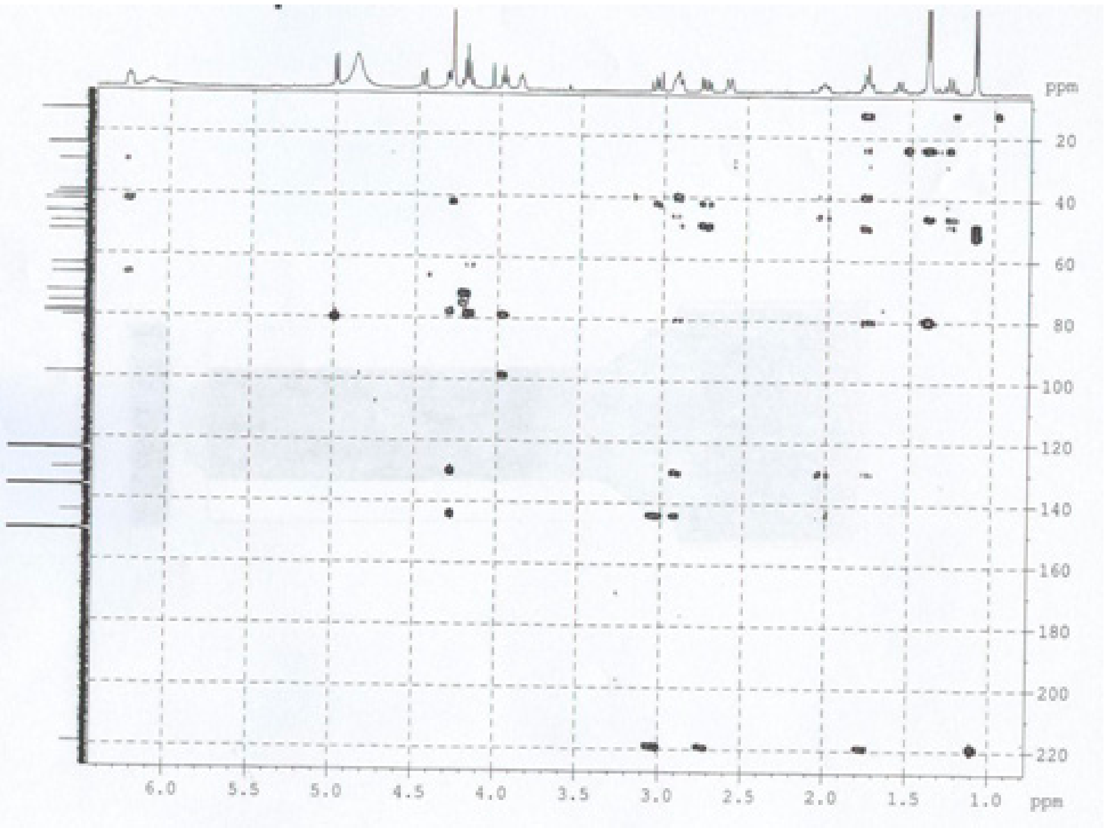

Figure S12 HMBC spectrum of compound **2**

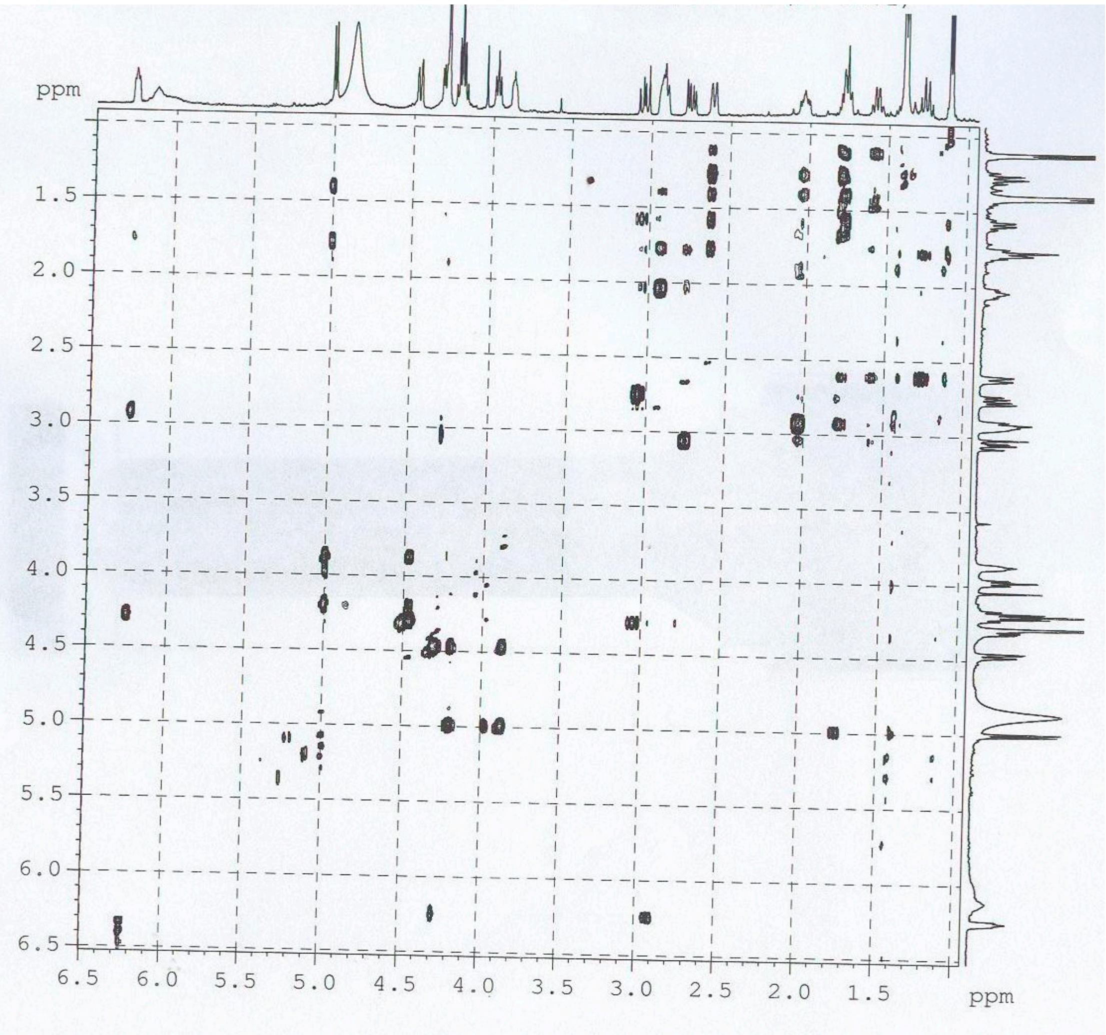

Figure S13 ROESY spectrum of compound **2**

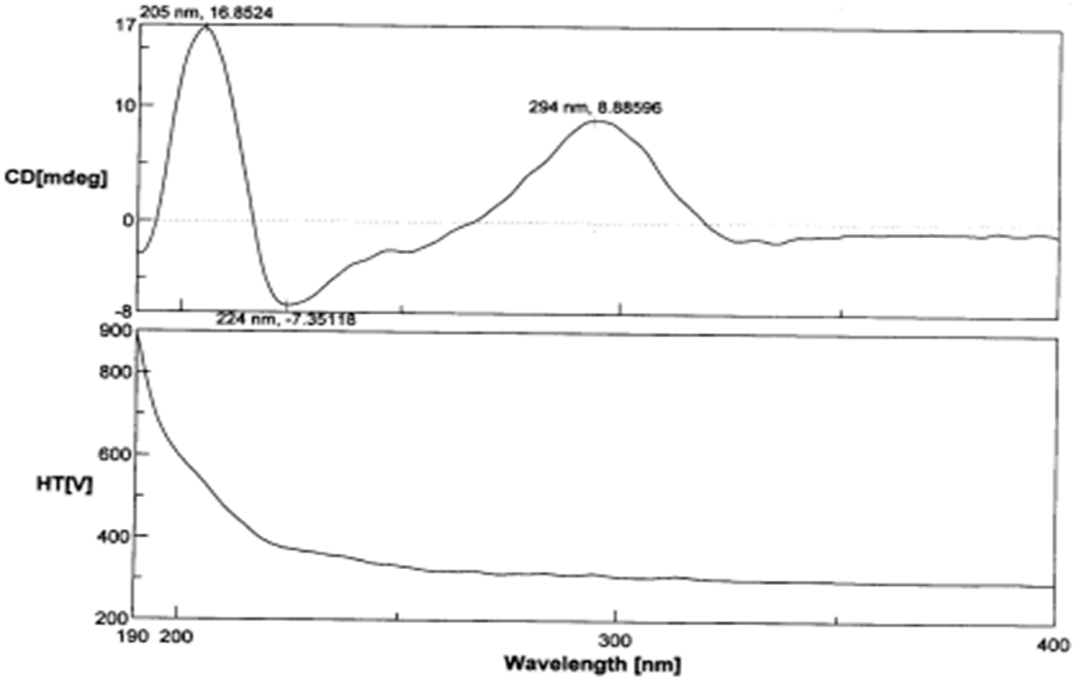

Figure S14 CD spectrum of compound **2**

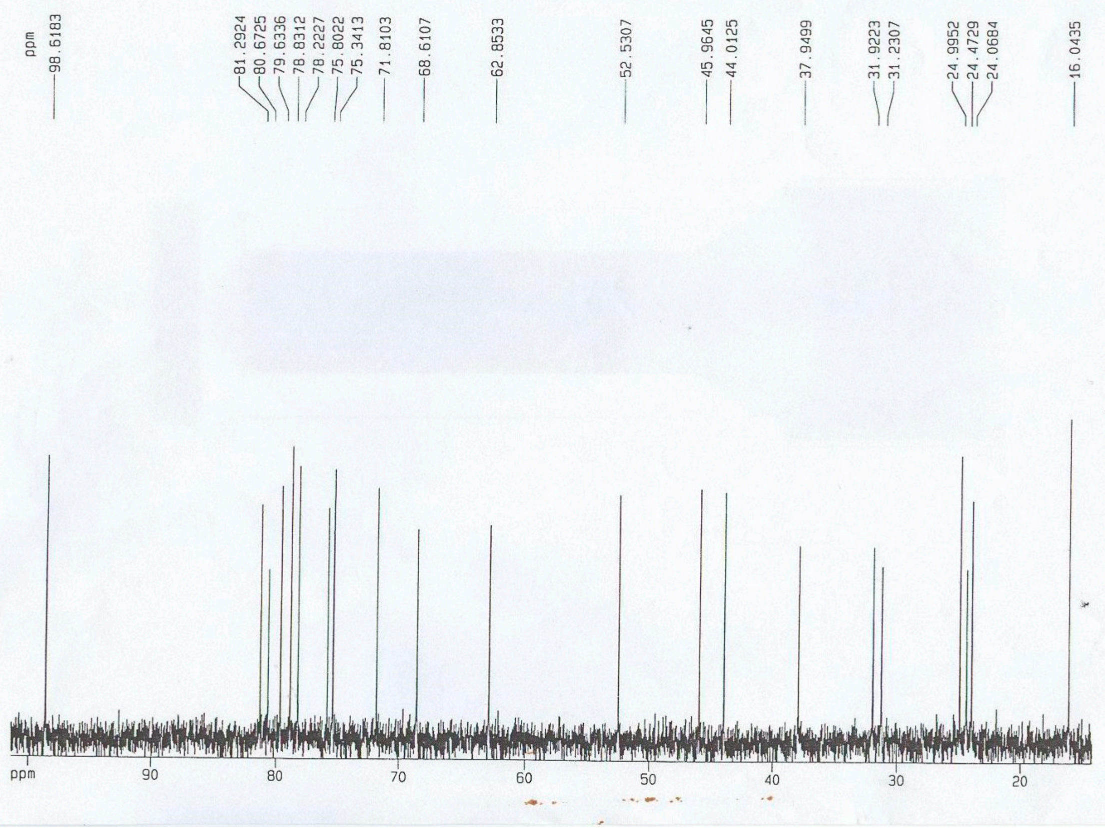

Figure S15 ^{13}C-NMR spectrum of compound **3**

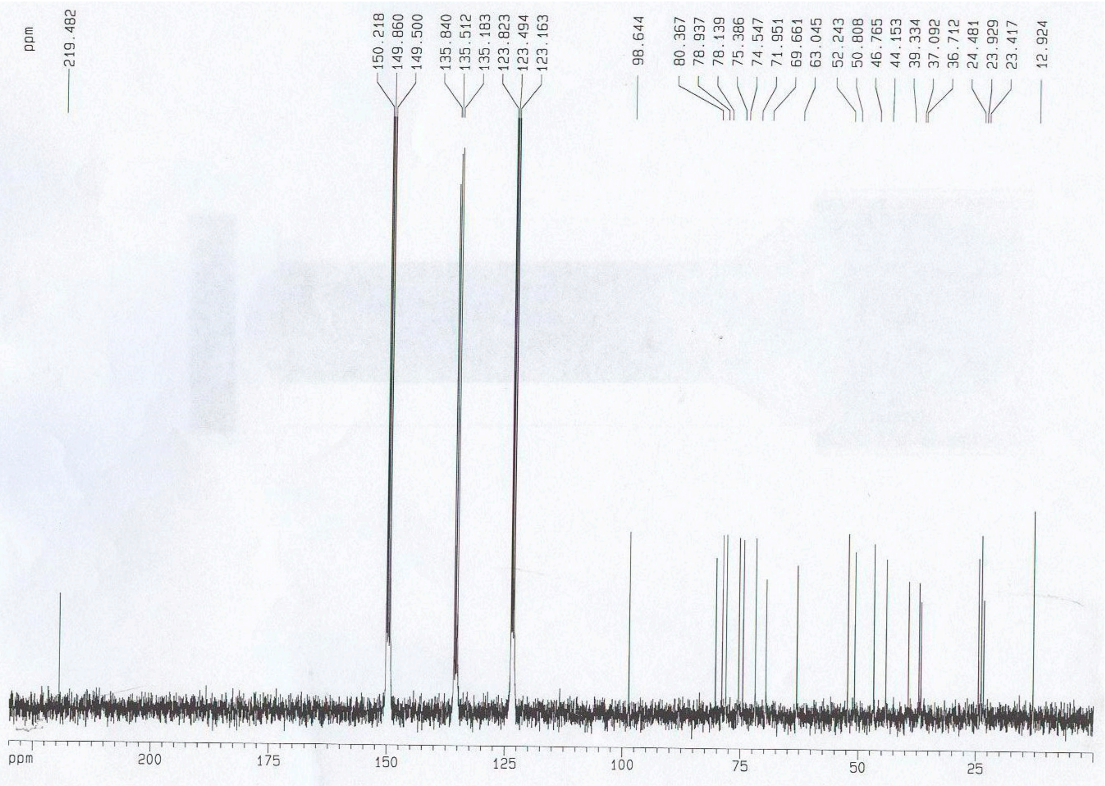

Figure S16 ^{13}C-NMR spectrum of compound **4**

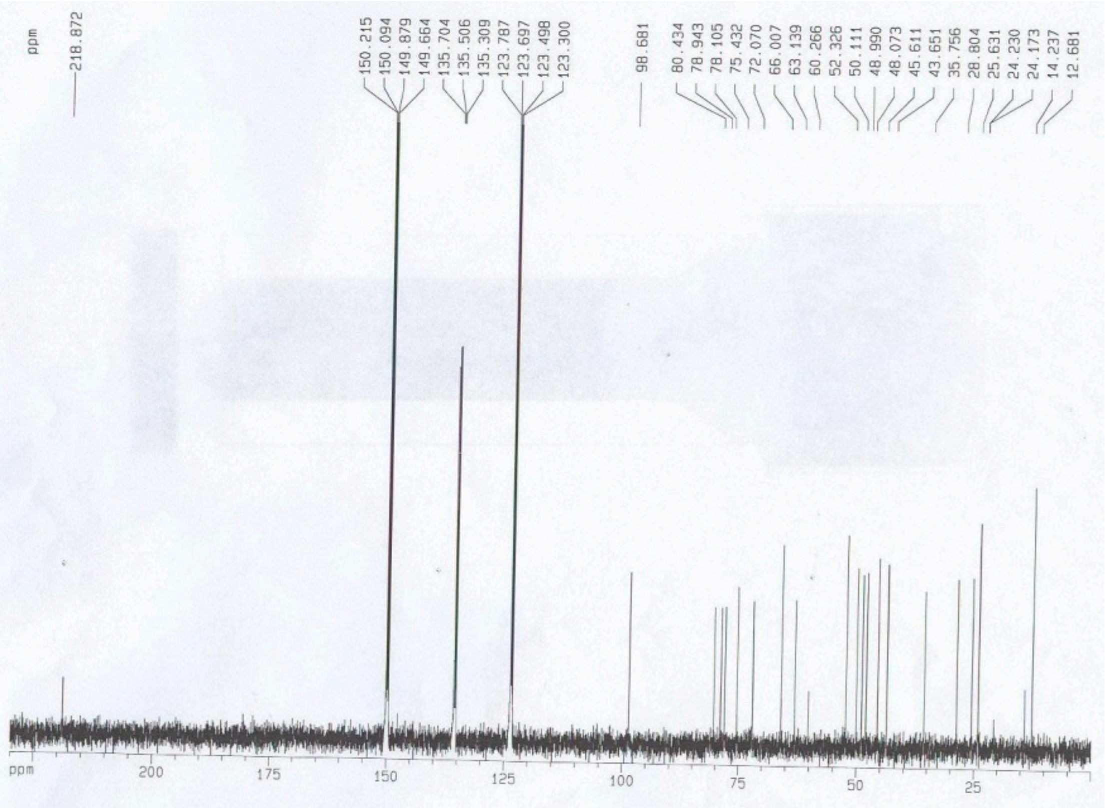

Figure S17 ^{13}C-NMR spectrum of compound **5**

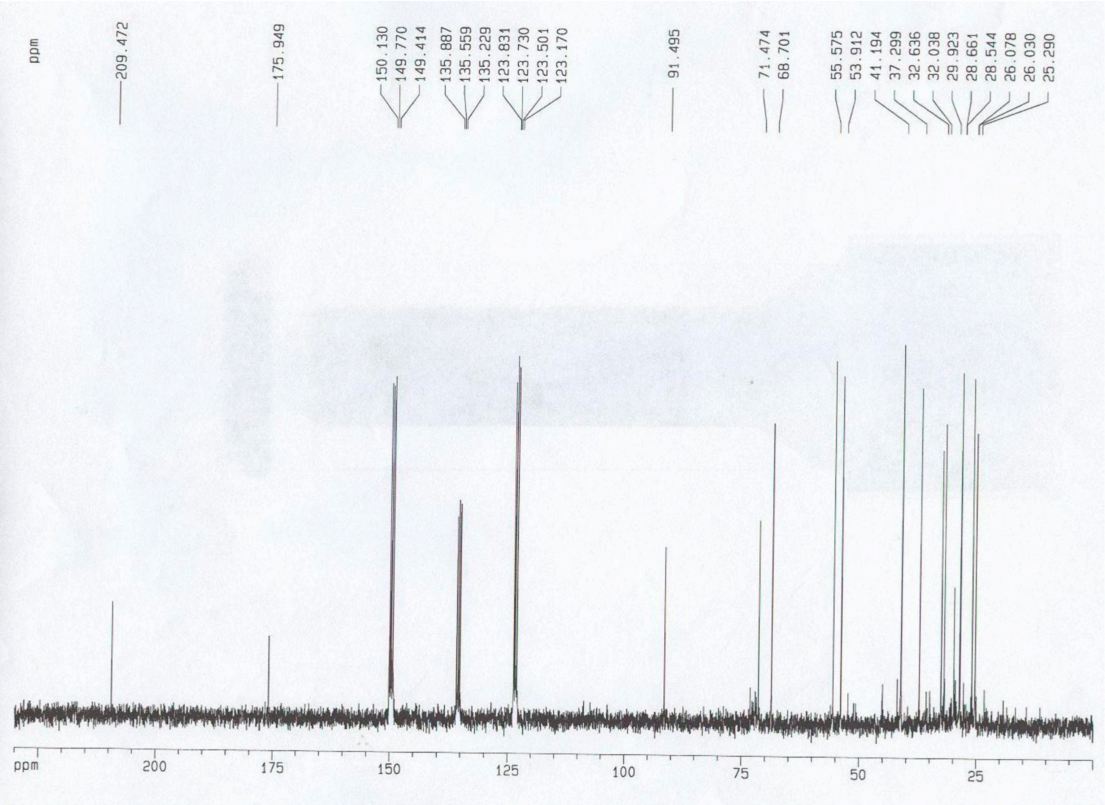

Figure S18 ^{13}C-NMR spectrum of compound **6**

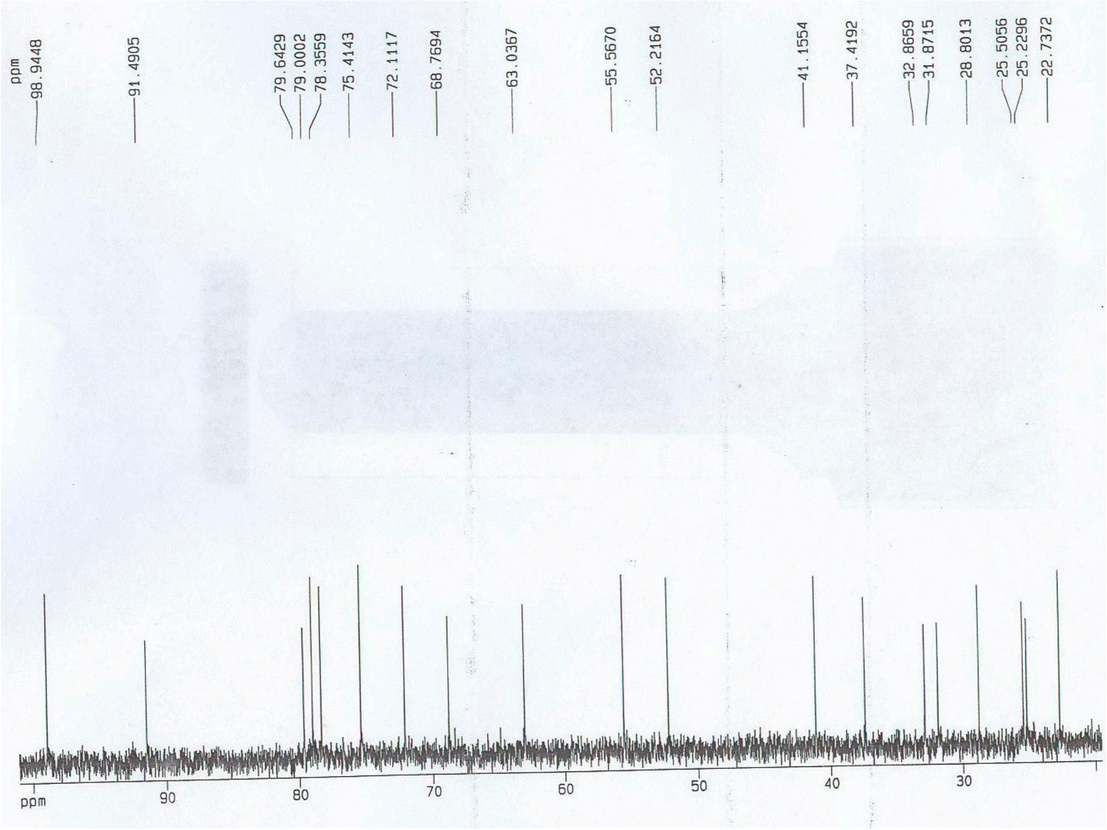

Figure S19 ^{13}C-NMR spectrum of compound **7**

Table S1. The top 50 potential drug targets of compound **1**

	***Z*-score**	**Fit**	**Name**	**Gene Symbol**	**Function**	**Idication**
**1**	4.15289	3.843	C-1-tetrahydrofolate synthase, cytoplasmic	MTHFD1	Nucleotide transport and metabolism	For nutritional supplementation, also for treating dietary shortage or imbalance.
**2**	3.05957	3.794	Protein S100-A9	S100A9	Expressed by macrophages in acutely inflammated tissues and in chronic inflammations. Seem to be an inhibitor of protein kinases. Also expressed in epithelial cells constitutively or induced during dermatoses. May interact with components of the intermedi	NONE
**3**	2.92923	3.761	Cathepsin F	CTSF	Involved in cysteine-type endopeptidase activity	NONE
**4**	2.91657	3.44	Cytidine deaminase	CDA	This enzyme scavenge exogenous and endogenous cytidine and 2-deoxycytidine for UMP synthesis.	NONE
**5**	2.71783	3.495	Uridine-cytidine kinase 2	UCK2	Nucleotide transport and metabolism	NONE
**6**	2.64053	3.564	Histone acetyltransferase PCAF	KAT2B	Involved in N-acetyltransferase activity	NONE
**7**	2.62491	3.619	Sulfotransferase family cytosolic 2B member 1	SULT2B1	Involved in sulfotransferase activity	NONE
**8**	2.58506	3.482	Phenylalanine-4-hydroxylase	PAH	NONE	Phenylketonuria;Sickle cell anemia;Vascular disease;Genetic disorder;Diabetic nephropathy;Hypertension;Ataxia;Peripheral arterial occlusive disease;Coronary artery disease

**9**	2.56743	3.506	Tryptase beta-2	TPSB2	Tryptase is the major neutral protease present in mast cells and is secreted upon the coupled activation-degranulation response of this cell type. Has an immunoprotective role during bacterial infection. Required to efficiently combat K.pneumoniae infecti	NONE
**10**	2.5397	3.522	ADP-ribosyl cyclase 2	BST1	Synthesizes cyclic ADP-ribose, a second messenger that elicits calcium release from intracellular stores. May be involved in pre-B-cell growth.	Hematological neoplasm;Multiple myeloma
**11**	2.52564	3.911	Thymidylate kinase	DTYMK	Catalyzes the conversion of dTMP to dTDP.	NONE
**12**	2.45288	3.583	Methionine aminopeptidase 2	MAP2	Removes the amino-terminal methionine from nascent proteins.	NONE
**13**	2.45033	3.504	Signal transducer and activator of transcription 1-alpha/beta	STAT1	Signal transducer and activator of transcription that mediates signaling by interferons (IFNs). Following type I IFN (IFN-alpha and IFN-beta) binding to cell surface receptors, Jak kinases (TYK2 and JAK1) are activated, leading to tyrosine phosphorylation	Respiratory disease;Asthma
**14**	2.37538	3.493	Glucosamine-6-phosphate isomerase	GNPDA1	Carbohydrate transport and metabolism	NONE
**15**	2.20187	4.096	Histidine triad nucleotide-binding protein 1	HINT1	Hydrolyzes adenosine 5-monophosphoramidate substrates such as AMP-morpholidate, AMP-N-alanine methyl ester, AMP-alpha- acetyl lysine methyl ester and AMP-NH2 (By similarity).	NONE
**16**	2.14521	3.384	Glycogen synthase kinase-3 beta	GSK3B	Involved in protein kinase activity	NONE

**17**	2.05188	3.66	Dihydroorotate dehydrogenase, mitochondrial	DHODH	NONE	NONE
**18**	1.97644	3.767	GMP reductase 2	GMPR2	Catalyzes the irreversible NADPH-dependent deamination of GMP to IMP. It functions in the conversion of nucleobase, nucleoside and nucleotide derivatives of G to A nucleotides, and in maintaining the intracellular balance of A and G nucleotides (PubMed:12009299, PubMed:12669231, PubMed:16359702, PubMed:22037469). Plays a role in modulating cellular differentiation (PubMed:12669231).	NONE
**19**	1.82894	3.266	Fructose-bisphosphate aldolase A	ALDOA	NONE	NONE
**20**	1.82601	3.446	Sorbitol dehydrogenase	SORD	NONE	Diabetic complication
**21**	1.68656	3.188	Superoxide dismutase [Mn], mitochondrial	SOD2	Destroys superoxide anion radicals which are normally produced within the cells and which are toxic to biological systems.	NONE
**22**	1.67957	3.174	3-phosphoinositide-dependent protein kinase 1	PDPK1	Phosphorylates and activates not only PKB/AKT, but also PKA, PKC-zeta, RPS6KA1 and RPS6KB1. May play a general role in signaling processes and in development (By similarity). Isoform 3 is catalytically inactive.	NONE

**23**	1.67755	3.797	GMP reductase 1	GMPR	Catalyzes the irreversible NADPH-dependent deamination of GMP to IMP. It functions in the conversion of nucleobase, nucleoside and nucleotide derivatives of G to A nucleotides, and in maintaining the intracellular balance of A and G nucleotides.	NONE
**24**	1.64325	4.018	Apoptotic protease-activating factor 1	APAF1	Oligomeric Apaf-1 mediates the cytochrome c-dependent autocatalytic activation of pro-caspase-9 (Apaf-3), leading to the activation of caspase-3 and apoptosis. This activation requires ATP. Isoform 6 is less effective in inducing apoptosis.	NONE
**25**	1.62737	3.824	Glucose-6-phosphate isomerase	GPI	Neurotrophic factor for spinal and sensory neurons.	NONE
**26**	1.62243	3.356	Hexokinase-1	HK1	NONE	NONE
**27**	1.58443	3.578	Glutathione S-transferase A3	GSTA3	Involved in glutathione transferase activity	For nutritional supplementation, also for treating dietary shortage or imbalance
**28**	1.52038	3.352	Matrix metalloproteinase-9	MMP9	May play an essential role in local proteolysis of the extracellular matrix and in leukocyte migration. Could play a role in bone osteoclastic resorption. Cleaves KiSS1 at a Gly-	Defects in MMP9 may be a cause of susceptibility to lumbar disk herniation (LDH) [MIM:603932]. LDH is the predominant cause of low-back pain and unilateral leg pain.
**29**	1.44093	3.386	GTPase HRas	HRAS	Ras proteins bind GDP/GTP and possess intrinsic GTPase activity.	NONE
**30**	1.42362	3.645	Peptidyl-prolyl cis-trans isomerase FKBP1A	FKBP1A	May play a role in modulation of ryanodine receptor isoform-1 (RYR-1), a component of the calcium release channel of skeletal muscle sarcoplasmic reticulum. There are four molecules of FKBP12 per skeletal muscle RYR. PPIases accelerate the folding of prot	NONE

**31**	1.40476	3.791	Protein-glutamine gamma-glutamyltransferase E	TGM3	Catalyzes the cross-linking of proteins and the conjugation of polyamines to proteins. It is responsible for the later stages of cell envelope formation in the epidermis and the hair follicle.	NONE
**32**	1.37708	3.734	Angiotensin-converting enzyme	ACE2	Converts angiotensin I to angiotensin II by release of the terminal His-Leu, this results in an increase of the vasoconstrictor activity of angiotensin. Also able to inactivate bradykinin, a potent vasodilator. Has also a glycosidase activity which releas	NONE
**33**	1.30646	3.36	Glutathione S-transferase Mu 2	GSTM2	Conjugation of reduced glutathione to a wide number of exogenous and endogenous hydrophobic electrophiles.	NONE
**34**	1.24774	3.435	Leukotriene A-4 hydrolase	LTA4H	Hydrolyzes an epoxide moiety of leukotriene A4 (LTA-4) to form leukotriene B4 (LTB-4). The enzyme also has some peptidase activity.	Inflammation;Cancer;Acute myelogenous leukemia;Myocardial infarction;Non-small-cell lung cancer;Solid tumor
**35**	1.21816	3.542	Pyruvate kinase isozymes R/L	PKLR	NONE	NONE
**36**	1.15182	3.356	ADP-ribosylation factor-like protein 5A	ARL5A	Involved in GTP binding	NONE
**37**	1.15044	2.952	BAG family molecular chaperone regulator 1	BAG1	Inhibits the chaperone activity of HSP70/HSC70 by promoting substrate release. Inhibits the pro-apoptotic function of PPP1R15A, and has anti-apoptotic activity. Markedly increases the anti-cell death function of BCL2 induced by various stimuli.	NONE

**38**	1.12543	2.956	Stromelysin-1	MMP3	Can degrade fibronectin, laminin, gelatins of type I, III, IV, and V; collagens III, IV, X, and IX, and cartilage proteoglycans. Activates procollagenase.	NONE
**39**	1.11831	3.874	Aldo-keto reductase family 1 member C2	AKR1C2	Works in concert with the 5-alpha/5-beta-steroid reductases to convert steroid hormones into the 3-alpha/5-alpha and 3-alpha/5-beta-tetrahydrosteroids. Catalyzes the inactivation of the most potent androgen 5-alpha-dihydrotestosterone (5-alpha-DHT) to 5-	NONE
**40**	1.10238	2.949	Cyclin-T1	CCNT1	Regulatory subunit of the cyclin-dependent kinase pair (CDK9/cyclin-T1) complex, also called positive transcription elongation factor B (P-TEFb), which is proposed to facilitate the transition from abortive to productive elongation by phosphorylating the	NONE
**41**	1.09507	2.964	Cathepsin G	CTSG	Serine protease with trypsin- and chymotrypsin-like specificity.	Connective tissue disease;Viral infection;Inflammation;HIV infection
**42**	1.06213	2.922	P-selectin	SELP	Ca(2+)-dependent receptor for myeloid cells that binds to carbohydrates on neutrophils and monocytes. Mediates the interaction of activated endothelial cells or platelets with leukocytes. The ligand recognized is sialyl-Lewis X. Mediates rapid rolling of	Sickle cell anemia;Inflammation;Transplant rejection;Myocardial infarction;Restenosis;Thrombosis
**43**	1.03062	2.995	Platelet glycoprotein Ib alpha chain	GP1BA	Involved in protein binding	NONE

**44**	1.02122	2.939	Oxysterols receptor LXR-beta	NR1H2	Orphan receptor. Binds preferentially to double-stranded oligonucleotide direct repeats having the consensus half-site sequence 5-AGGTCA-3 and 4-nt spacing (DR-4).	Lipid metabolism disorder;Atherosclerosis
**45**	1.0122	3.542	Beta-hexosaminidase beta chain	HEXB	Carbohydrate transport and metabolism	NONE
**46**	1.01201	2.916	Dipeptidyl peptidase 4	DPP4	Removes N-terminal dipeptides sequentially from polypeptides having unsubstituted N-termini provided that the penultimate residue is proline. Plays a role in T-cell activation.	Rheumatoid arthritis;Diabetes mellitus;Non-insulin dependent diabetes;Autoimmune disease;Immune disorder
**47**	1.00313	2.916	Thymidine phosphorylase	TYMP	May have a role in maintaining the integrity of the blood vessels. Has growth promoting activity on endothelial cells, angiogenic activity in vivo and chemotactic activity on endothelial cells in vitro.;Catalyzes the reversible phosphorolysis of thymidine. The produced molecules are then utilized as carbon and energy sources or in the rescue of pyrimidine bases for nucleotide synthesis.	Angiogenesis disorder;Cancer
**48**	0.993325	2.933	Complement factor D	CFD	Factor D cleaves factor B when the latter is complexed with factor C3b, activating the C3bbb complex, which then becomes the C3 convertase of the alternate pathway. Its function is homologous to that of C1s in the classical pathway.	Autoimmune disease;Reperfusion injury;Age related macular degeneration

**49**	0.978927	2.897	Caspase-3	CASP3	Involved in the activation cascade of caspases responsible for apoptosis execution. At the onset of apoptosis it proteolytically cleaves poly(ADP-ribose) polymerase (PARP) at a 216-Asp-	NONE
**50**	0.975398	2.94	Vascular endothelial growth factor receptor 2	KDR	Receptor for VEGF or VEGFC. Has a tyrosine-protein kinase activity. The VEGF-kinase ligand/receptor signaling system plays a key role in vascular development and regulation of vascular permeability. In case of HIV-1 infection, the interaction with extrace	Angiogenesis disorder;Melanoma;Cancer;Non-small-cell lung cancer;Renal cell carcinoma;Colorectal tumor;Ocular disease;Age related macular degeneration;Solid tumor

Table S2. The gene function analysis of top 50 candidate targets for compound **1**

		Gene Number	*P*-Value	Genes	Fold Enrichment	Bonferroni	Benjamini	FDR
	**Biological process**							
**1**	proteolysis	9	9.00E-05	DPP4, CFD, TPSB2, CASP3, MMP3, CTSG, LTA4H, CTSF, MMP9	6.045120000	0.055196482	0.018925245	0.018805276
**2**	oxidation-reduction process	8	0.001550296	MTHFD1, GMPR2, PAH, SORD, GMPR, AKR1C2, SOD2, DHODH	4.538378378	0.624311842	0.141761896	0.140863247
**3**	positive regulation of transcription from RNA polymerase II promoter	7	0.064524723	KAT2B, GSK3B, CCNT1, STAT1, NR1H2, HEXB, HRAS	2.396411825	1.000000000	1.000000000	0.995238095
**4**	negative regulation of apoptotic process	6	0.010231055	GSK3B, CASP3, BAG1, KDR, SOD2, MMP9	4.428659341	0.998480004	0.510384113	0.507148714
**5**	response to hypoxia	5	0.001572636	DPP4, PKLR, APAF1, CASP3, SOD2	9.762790698	0.629578740	0.141761896	0.140863247

**6**	response to drug	5	0.011774848	STAT1, CASP3, SORD, SOD2, DHODH	5.523684211	0.999432358	0.510384113	0.507148714
**7**	apoptotic process	5	0.082856627	APAF1, STAT1, CASP3, BAG1, S100A9	2.961552028	1.000000000	1.000000000	0.995238095
**8**	response to cadmium ion	4	5.13E-05	GPI, DTYMK, SORD, SOD2	53.734400000	0.031873060	0.018270541	0.018154722
**9**	canonical glycolysis	4	5.79E-05	GPI, PKLR, ALDOA, HK1	51.667692308	0.035882536	0.018270541	0.018154722
**10**	cellular response to insulin stimulus	4	0.001467686	KAT2B, PKLR, PDPK1, STAT1	17.446233766	0.604177870	0.141761896	0.140863247
	**Cell component**							
**1**	cytosol	27	3.83E-08	GPI, CDA, GSK3B, DTYMK, GMPR2, GMPR, HK1, TYMP, CASP3, BAG1, LTA4H, HRAS, GSTM2, PKLR, APAF1, PDPK1, STAT1, SORD, SULT2B1, FKBP1A, UCK2, GNPDA1, MTHFD1, GSTA3, PAH, ALDOA, S100A9	2.968615385	0.000003791	0.000001896	0.000001838
**2**	extracellular exosome	26	6.49E-09	CFD, GPI, HEXB, DPP4, HINT1, CTSG, LTA4H, CTSF, TGM3, GSTM2, PKLR, APAF1, SORD, GP1BA, SOD2, MMP9, SULT2B1, FKBP1A, ACE2, BST1, GNPDA1, MTHFD1, GSTA3, PAH, ALDOA, S100A9	3.371213091	0.000000642	0.000000642	0.000000623

**3**	cytoplasm	23	0.011173698	GPI, GSK3B, GSTM2, PDPK1, STAT1, NR1H2, GMPR2, GMPR, AKR1C2, GP1BA, DHODH, SELP, SULT2B1, FKBP1A, ACE2, HINT1, GNPDA1, MAP2, CASP3, BAG1, LTA4H, HRAS, TGM3	1.605331291	0.671238473	0.184366009	0.178779160
**4**	extracellular space	12	7.35E-04	SELP, CFD, GPI, ACE2, HEXB, MMP3, SORD, CTSG, CTSF, ALDOA, S100A9, MMP9	3.247037862	0.070246687	0.024269732	0.023534286
**5**	membrane	11	0.064958049	SELP, FKBP1A, DPP4, GPI, MTHFD1, PDPK1, HEXB, SORD, GP1BA, ALDOA, HRAS	1.822400000	0.998704974	0.714538540	0.692885857
**6**	extracellular region	10	0.026083119	CFD, CDA, ACE2, TPSB2, MMP3, KDR, CTSG, ALDOA, S100A9, MMP9	2.263850932	0.926942055	0.368889830	0.357711351
**7**	membrane raft	5	0.002257309	DPP4, ACE2, CASP3, KDR, HK1	8.846601942	0.200465996	0.055868403	0.054175421
**8**	GMP reductase complex	2	0.005370442	GMPR2, GMPR	364.480000000	0.413220716	0.106334749	0.103112484
**9**	I band	2	0.060081655	KAT2B, ALDOA	31.693913043	0.997832683	0.714538540	0.692885857
	**Molecular function**							
**1**	ATP binding	9	0.064446413	GSK3B, DTYMK, UCK2, PKLR, MTHFD1, APAF1, PDPK1, KDR, HK1	2.032494983	0.999999160	1.000000000	1.000000000
**2**	zinc ion binding	8	0.050491432	CDA, ACE2, NR1H2, MMP3, SORD, LTA4H, S100A9, MMP9	2.310487596	0.999981174	0.963927335	0.963927335

**3**	identical protein binding	7	0.020630994	DPP4, APAF1, STAT1, SORD, ALDOA, SOD2, MMP9	3.155327103	0.987447369	0.618929811	0.618929811
**4**	serine-type endopeptidase activity	6	8.38E-04	DPP4, CFD, TPSB2, MMP3, CTSG, MMP9	7.944000000	0.161493072	0.175625793	0.175625793
**5**	protein homodimerization activity	6	0.059542136	FKBP1A, DPP4, CDA, GSTM2, STAT1, HEXB	2.774958904	0.999997481	1.000000000	1.000000000
**6**	metallopeptidase activity	4	0.001672627	ACE2, MMP3, LTA4H, MMP9	16.672592593	0.296400284	0.175625793	0.175625793
**7**	catalytic activity	4	0.017211343	HINT1, GMPR2, TGM3, DHODH	7.183404255	0.973900728	0.602397016	0.602397016
**8**	kinase activity	4	0.032720072	GSK3B, DTYMK, PKLR, PDPK1	5.603651452	0.999075394	0.763468349	0.763468349
**9**	endopeptidase activity	3	0.010728738	ACE2, MMP3, MMP9	18.756666667	0.896191576	0.563258723	0.563258723
**10**	serine-type peptidase activity	3	0.014402129	DPP4, CFD, TPSB2	16.077142857	0.952472071	0.602397016	0.602397016

Table S3. KEGG pathway enrichment of top 50 candidate targets for compound **1**

	**Pathway**	**Gene Number**	***P*-Value**	**Genes**	**Fold Enrichment**	**Bonferroni**	**Benjamini**	**FDR**
**1**	Amino sugar and nucleotide sugar metabolism	4	0.003024544	GPI, GNPDA1, HEXB, HK1	13.33139535	0.349579433	0.180545582	0.175459791
**2**	Pyrimidine metabolism	5	0.00319425	CDA, DTYMK, UCK2, TYMP, DHODH	7.919640801	0.365113735	0.180545582	0.175459791
**3**	Thyroid hormone signaling pathway	5	0.005085791	KAT2B, GSK3B, PDPK1, STAT1, HRAS	6.955510617	0.51520299	0.180545582	0.175459791
**4**	Glycolysis / Gluconeogenesis	4	0.007731444	GPI, PKLR, ALDOA, HK1	9.550850399	0.667837537	0.219573008	0.213387853
**5**	Insulin signaling pathway	5	0.009618378	GSK3B, PKLR, PDPK1, HRAS, HK1	5.796258847	0.74650739	0.227634957	0.221222705
**6**	Hepatitis B	5	0.011393711	APAF1, STAT1, CASP3, HRAS, MMP9	5.516439455	0.803519972	0.231129557	0.224618865
**7**	Fructose and mannose metabolism	3	0.016076433	SORD, ALDOA, HK1	14.99781977	0.89988168	0.285356682	0.277318465

**8**	Bladder cancer	3	0.025676545	HRAS, MMP9, TYMP	11.70561543	0.975120114	0.388274793	0.377337475
**9**	Carbon metabolism	4	0.031157553	GPI, PKLR, ALDOA, HK1	5.6628936	0.988832693	0.388274793	0.377337475
**10**	Proteoglycans in cancer	5	0.032811954	PDPK1, CASP3, KDR, HRAS, MMP9	3.999418605	0.991239061	0.388274793	0.377337475
**11**	Endometrial cancer	3	0.039813053	GSK3B, PDPK1, HRAS	9.229427549	0.99687735	0.434881039	0.422630869
**12**	Hepatitis C	4	0.046932234	GSK3B, PDPK1, STAT1, HRAS	4.811330652	0.998914632	0.476026947	0.462617737
**13**	Prolactin signaling pathway	3	0.069422219	GSK3B, STAT1, HRAS	6.75958074	0.999963451	0.62318301	0.605628559
**14**	Biosynthesis of amino acids	3	0.071136431	PKLR, PAH, ALDOA	6.665697674	0.99997187	0.62318301	0.605628559
**15**	Metabolism of xenobiotics by cytochrome P450	3	0.074606417	GSTM2, GSTA3, AKR1C2	6.485543683	0.999983467	0.62318301	0.605628559

Table S4. The top 10 involved indications of top 50 candidate targets for compound **1**

**Term**	**Gene Number**	***P*-Value**	**Genes**	**Fold Enrichment**	**Bonferroni**	**Benjamini**	**FDR**
Type 2 diabetes	24	1.48E-07	CFD, GSK3B, GSTM2, APAF1, STAT1, HEXB, NR1H2, MMP3, GP1BA, SOD2, MMP9, TYMP, SELP, FKBP1A, DPP4, ACE2, MTHFD1, MAP2, CASP3, BAG1, KDR, CTSG, LTA4H, CTSF	3.078925505	9.99E-05	9.99E-05	9.57E-05
Lung cancer	13	5.37E-07	CDA, GSK3B, GSTM2, APAF1, STAT1, MMP3, AKR1C2, SOD2, MMP9, MTHFD1, GSTA3, CASP3, HRAS	6.181648215	3.63E-04	1.81E-04	1.74E-04
Atherosclerosis	9	2.59E-05	SELP, GSTM2, MTHFD1, NR1H2, MMP3, GP1BA, LTA4H, SOD2, MMP9	7.148744642	0.017337796	0.005829876	0.005579778
Colorectal cancer	9	9.39E-05	SELP, GSTM2, MTHFD1, CASP3, MMP3, KDR, SOD2, HRAS, MMP9	5.957287201	0.061512553	0.015870704	0.01518986

Esophageal adenocarcinoma	8	1.87E-04	CDA, GSK3B, MTHFD1, APAF1, CASP3, MMP3, KDR, MMP9	6.426854949	0.118916271	0.019789692	0.018940726
Chronic renal failure	12	2.02E-04	SELP, SULT2B1, FKBP1A, CDA, BST1, GSTM2, NR1H2, CASP3, MMP3, KDR, SOD2, MMP9	3.738938266	0.12759701	0.019789692	0.018940726
Plasma HDL cholesterol	7	2.05E-04	KAT2B, GSK3B, PDPK1, STAT1, NR1H2, HRAS, MMP9	7.927099703	0.129373351	0.019789692	0.018940726
Breast cancer	9	3.58E-04	KAT2B, GSK3B, GSTM2, MTHFD1, STAT1, KDR, AKR1C2, SOD2, MMP9	4.899236193	0.215088459	0.026904542	0.025750353
Angina	3	6.35E-04	MMP3, GP1BA, MMP9	76.90316206	0.348904425	0.042896268	0.041056043
Alzheimer's disease	7	9.58E-04	GSK3B, MTHFD1, NR1H2, MMP3, CTSG, SOD2, MMP9	5.927470949	0.476853106	0.053965217	0.051650141
